# Computational Analysis of an Autophagy/Translation Switch Based on Mutual Inhibition of MTORC1 and ULK1

**DOI:** 10.1371/journal.pone.0116550

**Published:** 2015-03-11

**Authors:** Paulina Szymańska, Katie R. Martin, Jeffrey P. MacKeigan, William S. Hlavacek, Tomasz Lipniacki

**Affiliations:** 1 College of Inter-Faculty Individual Studies in Mathematics and Natural Sciences, University of Warsaw, Warsaw, Poland; 2 Van Andel Institute, Grand Rapids, Michigan, United States of America; 3 Theoretical Division and Center for Nonlinear Studies, Los Alamos National Laboratory, Los Alamos, New Mexico, United States of America; 4 Institute of Fundamental Technological Research, Warsaw, Poland; Fondazione Edmund Mach, Research and Innovation Centre, ITALY

## Abstract

We constructed a mechanistic, computational model for regulation of (macro)autophagy and protein synthesis (at the level of translation). The model was formulated to study the system-level consequences of interactions among the following proteins: two key components of MTOR complex 1 (MTORC1), namely the protein kinase MTOR (mechanistic target of rapamycin) and the scaffold protein RPTOR; the autophagy-initiating protein kinase ULK1; and the multimeric energy-sensing AMP-activated protein kinase (AMPK). Inputs of the model include intrinsic AMPK kinase activity, which is taken as an adjustable surrogate parameter for cellular energy level or AMP:ATP ratio, and rapamycin dose, which controls MTORC1 activity. Outputs of the model include the phosphorylation level of the translational repressor EIF4EBP1, a substrate of MTORC1, and the phosphorylation level of AMBRA1 (activating molecule in BECN1-regulated autophagy), a substrate of ULK1 critical for autophagosome formation. The model incorporates reciprocal regulation of mTORC1 and ULK1 by AMPK, mutual inhibition of MTORC1 and ULK1, and ULK1-mediated negative feedback regulation of AMPK. Through analysis of the model, we find that these processes may be responsible, depending on conditions, for graded responses to stress inputs, for bistable switching between autophagy and protein synthesis, or relaxation oscillations, comprising alternating periods of autophagy and protein synthesis. A sensitivity analysis indicates that the prediction of oscillatory behavior is robust to changes of the parameter values of the model. The model provides testable predictions about the behavior of the AMPK-MTORC1-ULK1 network, which plays a central role in maintaining cellular energy and nutrient homeostasis.

## Introduction

In modern societies, aging is arguably the most unavoidable of all maladies. Encouragingly, a number of factors that mitigate the negative effects of aging and prolong lifespan and/or healthspan have been discovered [1]. Several of these factors, including caloric restriction, the small-molecule metabolite spermidine, and the immunosuppressive natural product rapamycin (also known as sirolimus), have been found to exert their longevity/anti-aging effects, at least in part, through upregulation of autophagy, an intracellular recycling/degradative process mediated by the endomembrane system and under the control of a complex regulatory system [2]. The process of autophagy provides nutrients during starvation and clears damaged organelles, such as mitochondria, as well as cytotoxic proteins, which may be misfolded and/or abnormally aggregated.

Besides playing a role in aging and aging-related diseases, autophagy serves important functions in immunity (e.g., through clearance of intracellular pathogens), protects against neurodegeneration (e.g., through clearance of protein aggregates), and acts as a double-edged sword in tumorigenesis (e.g., by providing nutrients to sustain cancer cells in harsh microenvironments and by contributing to cancer cell death through excessive degradation of cytoplasmic constituents) [3]. Thus, understanding the regulation of autophagy has importance for advancing basic understanding of cell biology, improving quality of life, and finding new treatments for an array of diseases.

A key negative regulator of autophagy is MTOR (mammalian or mechanistic target of rapamycin), a serine/threonine kinase that has been described as a master regulator of cell growth and metabolism. MTOR is responsible for processing numerous signals, including nutrient levels, such as leucine abundance, and stimulation from growth factors, such as insulin and insulin-like growth factor 1 (IGF1). In addition to regulating autophagy, MTOR is involved in regulating and coordinating related processes, such as protein synthesis. Protein synthesis and autophagy are connected in that a major outcome of autophagy is the liberation of amino acids for use in protein synthesis [4].

MTOR regulates autophagy by phosphorylating UNC-51-like kinase 1 (ULK1) and regulates protein synthesis by phosphorylating substrates such as ribosomal protein S6 kinase 1 (RPS6KB1, also known as S6K1) and eukaryotic initiation factor 4E (eIF4E)-binding protein 1 (EIF4EBP1, also known as 4E-BP1) [5]. The ability of MTOR to phosphorylate ULK1 and EIF4EBP1/RPS6KB1 (and related substrates) is dependent on association of MTOR with cofactors, particularly the regulatory associated protein of MTOR (RAPTOR or RPTOR). RPTOR is a scaffold protein that is capable of interacting simultaneously with MTOR and an MTOR substrate [6,7]. Indeed, one of its main functions is to recruit substrates to MTOR; through this function, RPTOR controls the specificity of MTOR. In general, a scaffold that colocalizes an enzyme with one of its substrates determines the effective *k*_cat_ and *K*_M_ of the enzyme-catalyzed reaction occurring on the scaffold [8]. The multicomponent protein complexes within which RPTOR and MTOR are colocalized, which characteristically contain certain other proteins, such as MLST8, are likely to have varying compositions but are collectively referred to by a single name, MTORC1 (MTOR complex 1) [5].

EIF4EBP1 is arguably the MTORC1 substrate that is most clearly involved in facilitating MTORC1-mediated regulation of protein synthesis. It is a translational repressor that is active when hypophosphorylated and inactive when phosphorylated by MTORC1 [9]. Thus, MTORC1-mediated phosphorylation of EIF4EBP1 has a positive effect on protein synthesis. MTORC1-mediated phosphorylation of RPS6KB1, and other substrates, also has a positive effect on protein synthesis. In contrast, MTORC1-mediated phosphorylation of ULK1 has a negative effect on autophagy, through the mechanisms discussed below. Thus, MTORC1 reciprocally regulates protein synthesis and autophagy.

As noted above, regulation of autophagy by MTORC1 occurs through phosphorylation of the serine/threonine kinase ULK1. Through its kinase activity, ULK1 initiates a cascade of signaling events that promotes the formation of autophagosomes, the double-membrane vesicles responsible for autophagic recycling through transport of cytoplasmic contents to lysosomes. ULK1 kinase activity depends on activating phosphorylation by the energy-sensing, multimeric kinase AMPK (AMP-activated protein kinase) [10], which mediates inhibitory phosphorylation of RPTOR in MTORC1 [11]. A key substrate of ULK1 is AMBRA1 (activating molecule in BECN1-regulated autophagy), which regulates the class III phosphoinositide-3 kinase complex containing the PIK3C3 (VPS34) catalytic subunit, which is essential for forming autophagosomes [12]. AMBRA1 directly interacts with BECN1 (Beclin-1), promoting BECN1 interaction with PIK3C3 and thereby increasing PIK3C3 kinase activity [13]. When phosphorylated by ULK1, AMBRA1 is liberated from the cytoskeleton and is able to properly localize the PIK3C3 complex to sites of autophagosome formation [12]. The effect of MTORC1-mediated phosphorylation of ULK1 is inhibition of ULK1 kinase activity and downstream processes, including AMBRA1 phosphorylation.

ULK1 kinase activity is inhibited by mTORC1 in two ways: by the kinase activity of MTOR (in MTORC1) and by the binding activity of RPTOR [10,14]. The principal direct consequences of MTORC1-mediated phosphorylation of ULK1 are inhibition of intrinsic ULK1 kinase activity and inhibition of ULK1 association with AMPK. The latter effect is also achieved through the apparently redundant mechanism of RPTOR binding to ULK1 in competition with AMPK. Because ULK1-AMPK interaction enables activating phosphorylation of ULK1 by AMPK, blocking ULK1-AMPK interaction has the effect of limiting ULK1 kinase activity. Interestingly, MTORC1-mediated inhibition of ULK1 has a positive reinforcing effect on this MTORC1 function. Positive feedback arises through relief of ULK1-mediated inhibition of MTORC1. When active, ULK1 inhibits MTORC1 through phosphorylation of RPTOR, which has the effect of limiting RPTOR-mediated recruitment of MTOR substrates to MTORC1 [15].

Through the mechanisms described above, the interactions of MTORC1 and ULK1 form a mutual repression circuit, in which each is both an enzyme and a substrate. This circuit is similar to the genetic toggle switch constructed by Gardner et al. [16] in their seminal synthetic biology study, but is more complicated and relatively fast acting. As is well known, mutual inhibition circuits are capable of exhibiting exotic dynamical behaviors, such as bistability, although factors beyond network wiring (viz., appropriate time scales and nonlinearities) are required to realize such behaviors.

Here, to determine if mutual inhibition of MTORC1 and ULK1 potentially contributes to control of autophagy and/or protein synthesis in an all-or-none manner or allows oscillations, we constructed a mechanistic model that captures interactions among the signaling proteins discussed above: the two key members of MTORC1, MTOR and RPTOR; the autophagy-initiating kinase ULK1; AMPK, which activates ULK1 and represses MTORC1; AMBRA1, a substrate of ULK1 critical for autophagy activation; and EIF4EBP1, which is taken as a representative of the downstream targets of MTORC1 that are relevant for MTORC1-mediated activation of protein synthesis. In the model, we consider two inputs or signals: the amount of rapamycin in complex with FKBP1A (FK506-binding protein 1A, also known as FKBP12), which allosterically inhibits MTORC1 kinase activity [17,18]; and the cellular energy level, or more precisely the intrinsic AMPK kinase activity or level of phosphorylation of the α subunit of AMPK (at T172 in isoform PRKAA2 and at T183 in isoform PRKAA1), which increases as the cellular AMP:ATP ratio increases [19]. As a simplification, the mechanism responsible for energy sensing by AMPK is not included in the model, and intrinsic AMPK kinase activity level is taken as an adjustable input parameter. Similarly, we take the level of FKBP1A-bound rapamycin as an adjustable input parameter, considering FKBP1A implicitly. The model incorporates site-specific details that are known about the protein-protein interactions of interest. In other words, the model explicitly considers specific sites of phosphorylation and linear motifs and protein domains responsible for binding and catalytic interactions of interest.

Analysis of the model indicates that bistability can arise from mutual inhibition of MTORC1 and ULK1 together with multisite (ULK1-mediated) phosphorylation of RPTOR. Analysis of the model also indicates that oscillations in MTORC1 activity can be generated as a consequence of slow negative feedback from ULK1 to AMPK, depending on the stress inputs, which affect the kinase activities of AMPK and MTORC1. According to the model, oscillations in MTORC1 activity can drive alternating periods of mutually exclusive autophagy and protein synthesis. A sensitivity analysis indicates that model-predicted behaviors are robust to parameter uncertainties, meaning that qualitative behaviors are maintained over a large region of parameter space around the parameter values presented in Table 1. Note that we will occasionally refer to the parameter values of Table 1 as the nominal parameter values. Oscillations in MTORC1 activity, or the effects of these oscillations, are potentially observable, and their detection would provide strong support for our model.

**Table 1:** Parameter values.

**Parameter**	**Description/comment and units**
Concentrations, copy number per cell	
[MTOR] = 2×10^4^	Assumed amount of MTOR available to interact with ULK1
[RPTOR] = 2×10^4^	RPTOR abundance was set to equal the abundance of MTOR
[ULK1] = 1×10^4^	Measured abundance of ULK1
[rapamycin*]	Adjustable input (an independent variable of the model)
[AMPK*]	Adjustable input (an independent variable of the model)
[EIF4EBP1] = 1×10^4^	EIF4EBP1 was introduced to measure MTORC1 activity
[AMBRA1] = 1×10^4^	AMBRA1 was introduced to measure ULK1 activity
Rate constants for (bimolecular) association reactions, (molecules/cell)^−1^s^−1^	
*a*_1_ = 10^−3^	Rapamycin* binds MTOR, Equation (1)
*a*_2_ = 10^−3^	RPTOR binds MTOR, Equation (2)
*a*_3_ = 10^−3^	RPTOR binds ULK1, Equation (3A)
*a*_4_ = 10^−5^	RPTOR binds EIF4EBP1, Equation (4A)
*a*_5_ = 10^−5^	AMPK binds ULK1, Equation (5A)
Rate constants for (unimolecular) dissociation reactions, s^−1^	
*d*_1_ = 10^−2^	Rapamycin* releases MTOR, Equation (1)
*d*_2_ = 10^−1^	RPTOR releases MTOR, Equation (2)
*d*_3_ = 10^−1^	RPTOR slowly releases ULK1, Equation (3B)
*d*_3,max_ = 10	RPTOR quickly releases ULK1, Equation (3C)
*d*_4_ = 1	RPTOR releases EIF4EBP1, Equation (4B)
*d*_5_ = 10	AMPK releases ULK1, Equation (5B)
Rate constants for (pseudo first-order) phosphorylation reactions, s^−1^	
*p*_1_ = 10	MTORC1 phosphorylates ULK1, Equation (6)
*p*_2_ = 10	MTORC1 phosphorylates EIF4EBP1, Equation (7)
*p*_3_ = 10	ULK1 phosphorylates S792 in RPTOR, Equation (8A)
*p*_4_ = 10	ULK1 phosphorylates S855/S859 in RPTOR, Equation (8B,C)
*p*_7_ = 10	AMPK phosphorylates S317 in ULK1, Equation (11A)
*p*_8_ = 10	AMPK phosphorylates S778 in ULK1, Equation (11B)
Rate constants for (pseudo second-order) phosphorylation reactions, (molecules/cell)^−1^s^−1^	
*p*_5_ = 10^−4^	ULK1 phosphorylates AMBRA1, Equation (9)
*p*_6_ = 10^−6^	ULK1 phosphorylates AMPK, Equation (10)
*p*_9_ = 0 or small enough	AMPK phosphorylates RPTOR, Equation (12)
Rate constants for (pseudo first-order) dephosphorylation reactions, s^−1^	
*u*_1_ = 10^−3^	Dephosphorylation of S792 in RPTOR, Equation (15A)
*u*_2_ = 10^−4^	Dephosphorylation of inhibitory sites in AMPK, Equation (17)
*u*_0_ = 10^−2^	All other sites, Equations (13), (14), (15B,C), (16), (18A,B), and (19)

## Results

### Model

**Fig 1:**
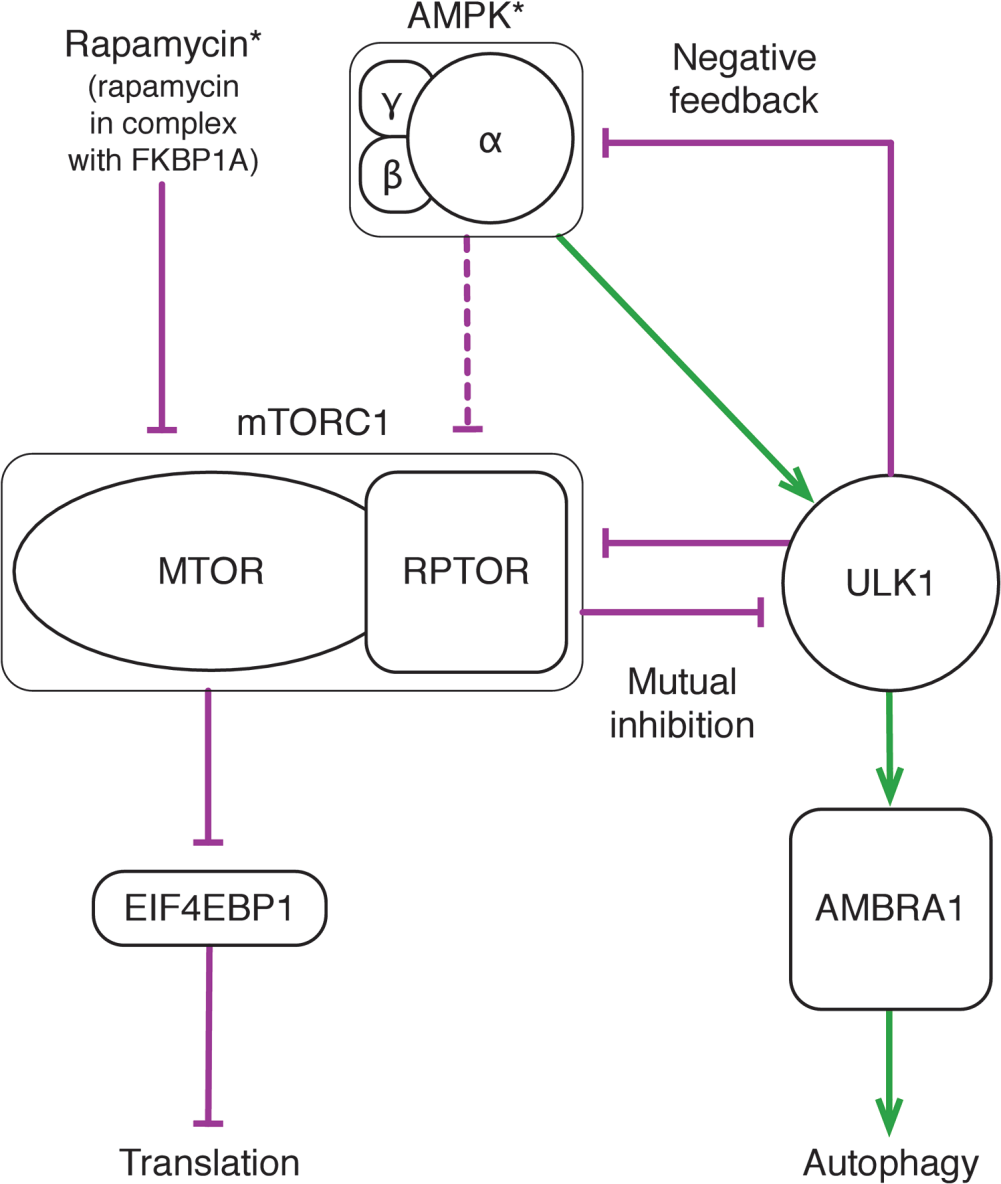
Schematic overview of the model. This diagram depicts the proteins considered in the model and regulatory influences among these proteins, as well as model inputs and outputs. Two inputs are considered: the level of rapamycin in complex with FKBP1A and therefore competent to inhibit MTORC1 (i.e., the level of rapamycin*) and the level of AMPK with activating phosphorylation in its kinase domain (i.e., the level of AMPK*). We note that AMPK activity is limited by the level of AMPK* and also the level of inhibitory phosphorylation of AMPK by ULK1. Thus, AMPK* does not necessarily correspond to the level of active AMPK. AMPK negatively regulates MTORC1 and positively regulates ULK1. A dashed arrow is used to represent the negative influence of AMPK on MTORC1 because this influence is not considered until the “Sensitivity analysis” section. Key regulatory influences considered in the model are mutual inhibition of MTORC1 and ULK1 and (slow) negative feedback from ULK1 to AMPK. Two outputs are considered: the level of phosphorylation of EIF4EBP1, a repressor of translation, and the level of phosphorylation of AMBRA1, which is involved in activating autophagy. We consider phosphorylation of EIF4EBP1 to be indicative of a translation state and phosphorylation of AMBRA1 to be indicative of an autophagy state.

On the basis of a literature survey and with the goal of investigating the possibility of bistable switching between translation and autophagy, we constructed a computational model that accounts for mutual inhibition of MTORC1 and ULK1 (Fig. 1). The model predicts system-level dynamics on the basis of principles of chemical kinetics (e.g., the law of mass action). The mechanistic resolution of the model is such that it tracks the time-dependent states of functional components of proteins of interest. In other words, the model tracks the occupancy of particular binding sites and the phosphorylation status of particular serine and threonine residues.

**Fig 2:**
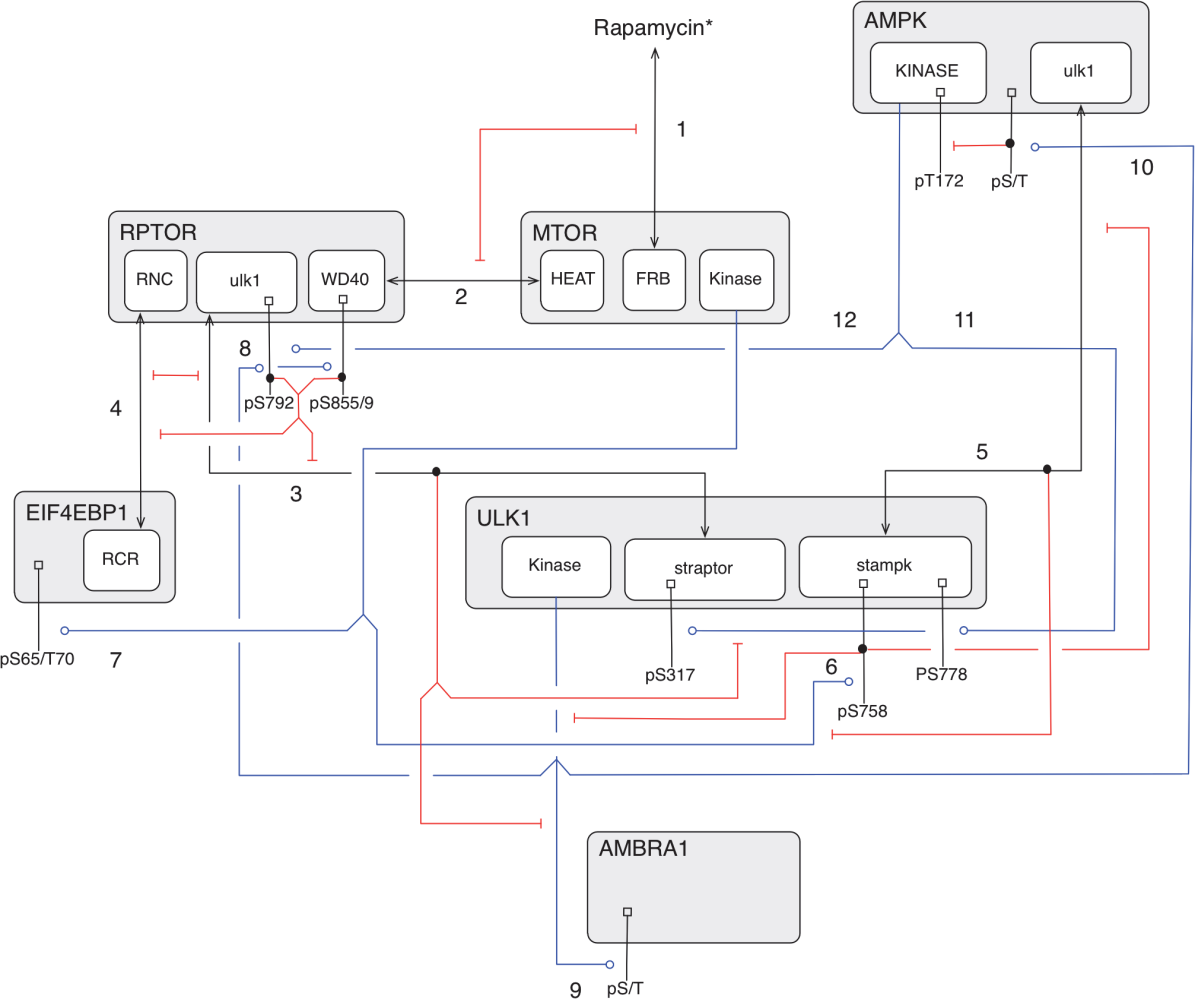
Illustration of site-specific details captured in the model. Nested boxes represent functional components of proteins. Black lines attached to small square boxes at one end and text labels at the other end indicate sites of serine/threonine phosphorylation. Blue lines that end with a circle indicate substrates of kinases. Black lines that begin and end with arrowheads represent direct binding interactions. Red lines with flat arrowheads indicate inhibitory effects of either serine/threonine phosphorylation reactions or direct binding interactions. Numbers next to arrows refer to sets of rules in the model (see Materials and Methods). The numbering of amino acid residues is consistent with UniProt entries for human proteins; the site of activating phosphorylation in AMPK is T172 (T183) in the PRKAA2 (PRKAA1) isoform of the AMPK α subunit. The following abbreviations are used for names of protein components: RNC, RAPTOR N-terminal conserved domain; WD40, solenoid protein domain consisting of WD40 repeats; HEAT, solenoid protein domain consisting of HEAT repeats; FRB, FKBP12-rapamycin binding domain; and RCR, RAPTOR crosslinking region. In the model, we consider several undefined regions within proteins; these regions are represented by names having all lowercase letters, which refer to binding partners. For further information, see Materials and Methods.

The model was formulated in terms of rules for protein-protein interactions (see Materials and Methods). The interactions represented by rules and included in the model are illustrated in the diagram of Fig. 2, which is drawn in accordance with recommended guidelines [20]. In building the model, we focused on including and specifying rules for interactions between AMPK, ULK1, and two key components of MTORC1, namely RPTOR and MTOR. (Other components of MTORC1, as well as proteins that localize ULK1 and AMBRA1 to membranes, are implicit in the model.) We further considered interactions between MTORC1 and EIF4EBP1 and ULK1 and AMBRA1, which are important for activating translation and autophagy, respectively. However, as a simplification, we do not consider positive feedback from AMBRA1 to ULK1 [21]. The phosphorylation levels of EIF4EBP1 and AMBRA1 are treated as outputs of the model and as markers for translation and autophagy. The model includes, in a simplified manner, sensing of rapamycin by MTOR and cellular energy level by AMPK. These processes depend on factors implicit in the model, such as FKBP1A, the cellular AMP:ATP ratio, and the protein kinase STK11 (also known as LKB1), which is responsible for activating phosphorylation of AMPK [22]. The amount of rapamycin competent to interfere with MTORC1 assembly and the level of intrinsic AMPK activity (i.e., the extent of activating phosphorylation of the kinase domain) are inputs of the model. We will refer to FBKP1A-bound rapamycin as rapamycin* and the phosphoform of AMPK with activating phosphorylation in the kinase domain as AMPK*. In the Materials and Methods section, we identify notable simplifications and limitations of the model. These issues are revisited in the Discussion section.

The mechanistic details depicted in Fig. 2 underlie a relatively simple network of stimulatory and inhibitory influences (Fig. 1). Arrows representing stimulation and inhibition are included in Fig. 2 to indicate how direct binding interactions and phosphorylation of particular serine and threonine residues influence binding and catalytic activities. At the core of the regulatory network depicted in Fig. 1 is the mutual repression circuit involving MTORC1 and ULK1. In the model, the inhibitory influences in this circuit arise from binding of RPTOR to ULK1, MTORC1-mediated phosphorylation of S758 in ULK1 [10,14,23], and ULK1-mediated phosphorylation of S792, S855 and S859 in RPTOR [15] (Fig. 2). Another notable feature of the regulatory network depicted in Fig. 1 is negative feedback from ULK1 to AMPK, which arises from ULK1-mediated phosphorylation of serine and threonine residues in a region of the α subunit of AMPK outside the kinase domain (Fig. 2) [24]. It should be noted that the form of AMPK with activating phosphorylation in the kinase domain, AMPK*, may exist in either an inhibited form (because of phosphorylation mediated by ULK1) or an uninhibited, active form.

Parameter values of the model, which were set as described in the Materials and Methods section, are summarized in Table 1. The parameter values are also given in S1 File in the Supporting Information, which is a complete, executable specification of the model.

### Bistability

**Fig 3:**
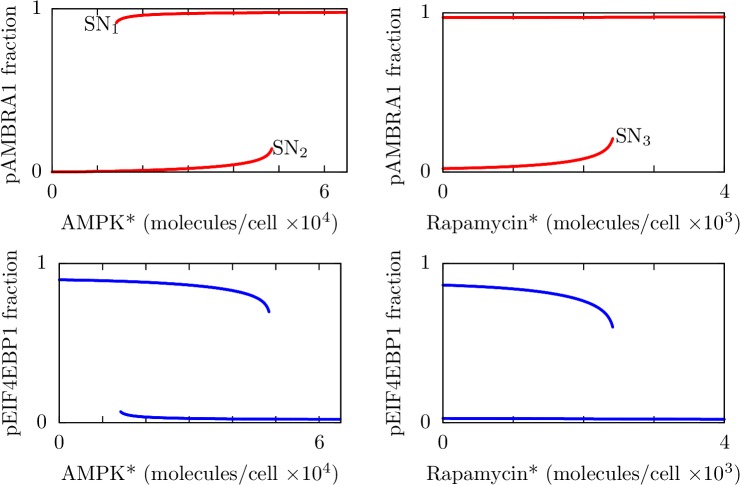
Results from bifurcation analysis of the system without negative feedback from ULK1 to AMPK. Each panel is a one-dimensional bifurcation diagram showing stable steady-state levels of phosphorylated AMBRA1 (red curves, top panels) or phosphorylated EIF4EBP1 (blue curves, bottom panels) as a function of the level of AMPK* (left panels) or the level of rapamycin* (right panels). Thus, we took the inputs of the model as our bifurcation parameters. For the left panels, the abundance of rapamycin* is fixed at zero. For the right panels, the abundance of AMPK* is fixed at 30,000 copies per cell. For all panels, the parameters considered in Table 1 are held fixed at their nominal values. The labels SN_1_, SN_2_, and SN_3_ indicate saddle node bifurcation points. Bifurcation analysis was performed numerically (see Materials and Methods).

In our parameterization of the model (Table 1), the negative feedback from ULK1 to AMPK (Fig. 1) is slow compared to other interactions. Thus, on short time scales, the system behaves as if the negative feedback is absent. For this reason, we first analyzed system behavior in the absence of negative feedback (i.e., without ULK1-mediated inhibitory phosphorylation of AMPK). As described in Materials and Methods, we found stable steady states of the system through simulation for different values of bifurcation parameters. The results are summarized in Fig. 3, where curves mark stable steady states. As can be seen, the system without negative feedback exhibits bistability over a broad range of each of the bifurcation parameters, the levels of AMPK* (left panels) and rapamycin* (right panels). It should be noted that, for the scenario under consideration, AMPK is always active when the kinase domain in the α subunit is phosphorylated (Fig. 2). In other words, for this scenario, AMPK* is equivalent to active AMPK.

The bifurcation diagrams of Fig. 3 indicate that the levels of translation and autophagy in a cell may be inversely related. When the level of AMPK* is chosen as a bifurcation parameter, there are two regions of monostability in parameter space (left panels). At low levels of AMPK*, AMBRA1 phosphorylation, which we take as a measure of the level of autophagy in a cell, is low (top panel), whereas EIF4EBP1 phosphorylation, which we take as a measure of the level of translation in a cell, is high (bottom panel). In contrast, at high levels of AMPK*, AMBRA1 phosphorylation is high (top panel), whereas EIF4EBP1phosphorylation is low (bottom panel). When the level of rapamycin* is chosen as a bifurcation parameter, there is only one region of monostability in parameter space at high levels of rapamycin* (right panels). In this region, AMBRA1 phosphorylation is high (top panel), whereas EIF4EBP1 phosphorylation is low (bottom panel). Where there is bistability (i.e., two stable steady states at a given value of a bifurcation parameter), high (or low) phosphorylation levels of AMBRA1 and EIF4EBP1 are mutually exclusive, which can be seen by imagining slow continuous changes of the relevant bifurcation parameter along the curves marking stable steady states, from far left to right and then from far right to left. For example, for the left panels of Fig. 3, AMBRA1 phosphorylation is low and EIF4EBP1 phosphorylation is high as AMPK* level increases from zero until the bifurcation point labeled SN_2_ (a saddle node bifurcation) is reached. At this point, AMBRA1 phosphorylation jumps to a high level and EIF4EBP1 phosphorylation jumps to a low level.

Thus, given our model structure (Figs. 1 and 2), parameter values (Table 1), and lack of negative feedback from ULK1 to AMPK, the results of Fig. 3 indicate that high (or low) phosphorylation levels of AMBRA1 and EIF4EBP1 are mutually exclusive over all regions of the parameter space of inputs considered. This type of behavior is a known consequence of mutual repression. Thus, mutual inhibition of MTORC1 and ULK1 may cause translation (promoted by phosphorylation of EIF4EBP1) and autophagy (promoted by phosphorylation of AMBRA1) to be mutually exclusive for the special case under consideration (i.e., no negative feedback from ULK1 to AMPK). The translation and autophagy states are characterized by distinct patterns of phosphorylation of RPTOR and ULK1 (Fig. A in S2 File in the Supporting Information). These patterns are such that, in the translation state, MTORC1 is active, and in the autophagy state, ULK1 is active.

An aspect of the qualitative system behavior revealed in Fig. 3 is that phosphorylation of AMBRA1 (which we take as a measure of autophagy level) cannot return to a low level once a high level has been induced by rapamycin*, even if rapamycin* is entirely cleared from the system (top right panel). Thus, at least for the parameter values considered (including a setting of AMPK* level at 30,000 copies per cell for the right panels of Fig. 3), termination of rapamycin-induced autophagy appears to require an additional regulatory mechanism beyond the mechanisms considered in the analysis of Fig. 3. A candidate mechanism for termination of autophagy within the scope of our model is the negative feedback from ULK1 to AMPK [24], because ULK1 activity (which promotes autophagy) depends on AMPK activity. Below we investigate this possibility.

### Oscillations

**Fig 4:**
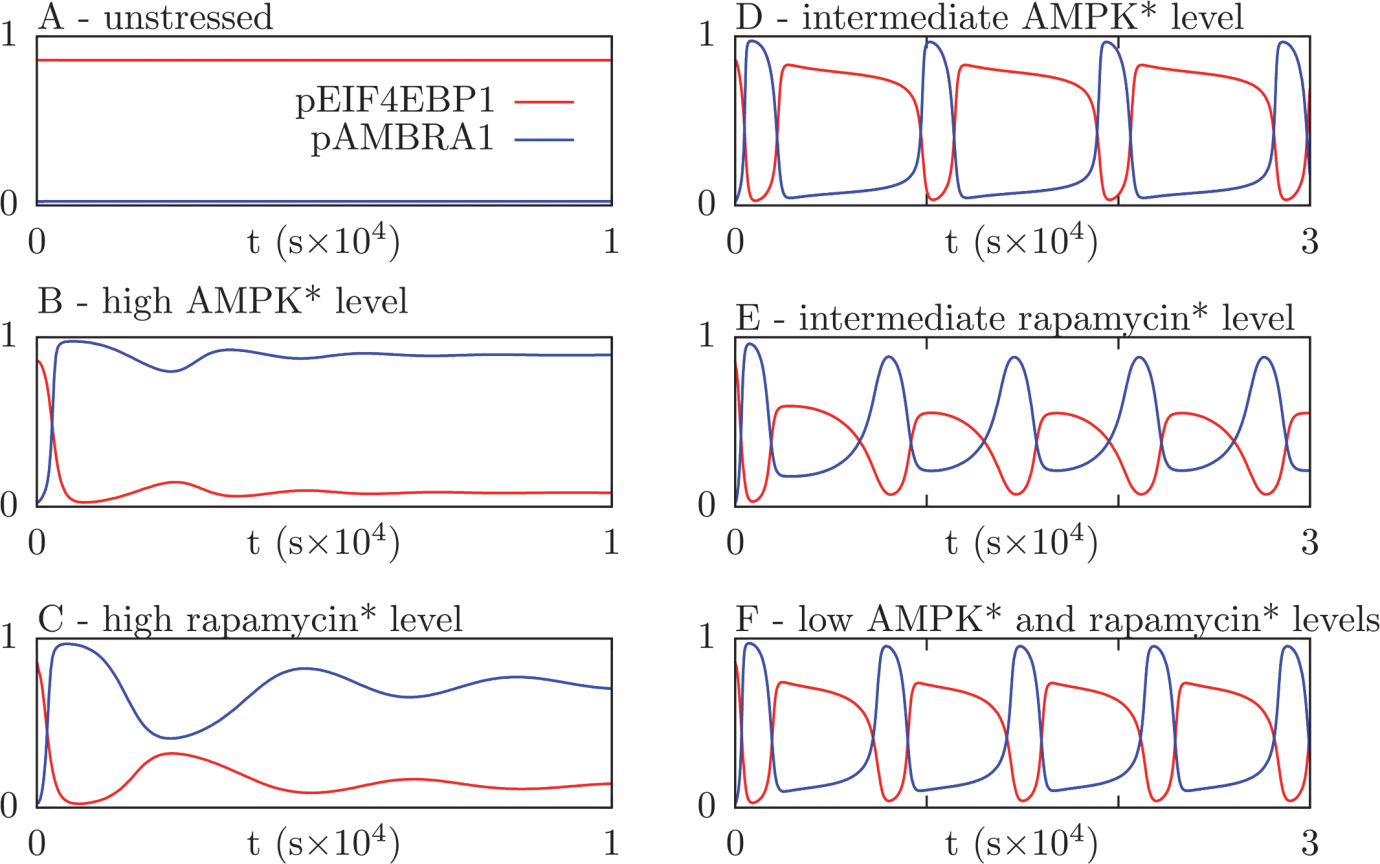
Time-dependent levels of AMBRA1 and EIF4EBP1 phosphorylation after various stimuli. In all panels, red curves indicate the fraction of total AMBRA1 phosphorylated by ULK1 and blue curves indicate the fraction of total EIF4EBP1 phosphorylated by MTORC1. In each panel, we simulate a cellular response to stress; the simulation begins at an “unstressed” steady state where the level of rapamycin* is zero and the level of AMPK* is 30,000 copies per cell. The stresses introduced at time *t* = 0 are as follows: (A) no stimulus (i.e., rapamycin* and AMPK* remain at their starting levels), (B) an increase in AMPK* level to 150,000 copies per cell, (C) an increase in rapamycin* level to 9,000 copies per cell, (D) an increase in AMPK* level to 90,000 copies per cell, (E) an increase in rapamycin* level to 6,000 copies per cell, and (F) increases in the rapamycin* level to 3,000 copies per cell and AMPK* level to 60,000 copies per cell.

In this section, we analyze consequences of negative feedback from ULK1 to AMPK. In Fig. 4, we show responses of this system to various stimuli. Fig. 4A shows phosphorylation levels of AMBRA1 and EIF4EBP1 under an unstressed condition, meaning a condition without rapamycin* and with a baseline level of AMPK*. (Recall that AMPK* is the form of AMPK with activating phosphorylation in the kinase domain.) Stressed conditions, arising from perturbations of the unstressed condition, are considered in Fig. 4B and 4C. The stress considered in Fig. 4B is a high level of AMPK*, which can be caused by energy depletion in the form of a low AMP:ATP ratio. The stress considered in Fig. 4C is presence of a large amount of rapamycin*. As can be seen, each stress is sufficient to induce persistent autophagy and repression of translation, as measured by a high level of AMBRA1 phosphorylation and a low level of EIF4EBP1 phosphorylation. In Fig. 4D–4F, responses to less severe stresses are considered. In these cases, there are high-amplitude oscillations in the phosphorylation levels of AMBRA1 and EIF4EBP1 that generate alternating periods of autophagy and translation. Thus, the model predicts that a tonic high level of autophagy will be generated in response to severe stresses, but pulses of autophagy will be generated in response to moderate stresses.

**Fig 5:**
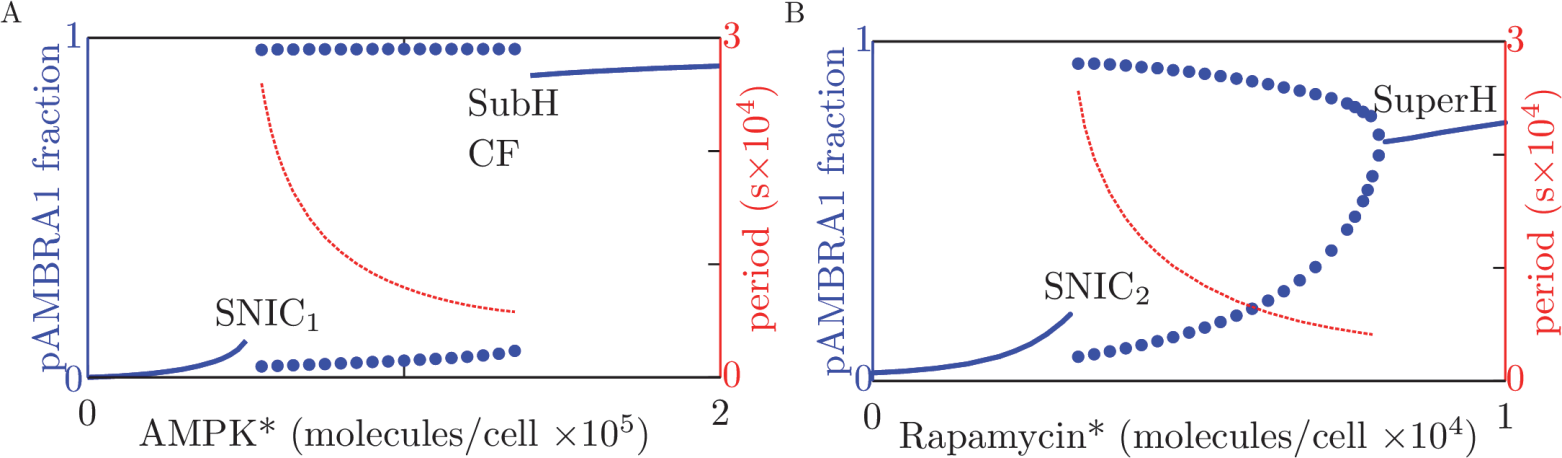
Results from bifurcation analysis of the system with negative feedback from ULK1 to AMPK. The solid blue curves indicate stable steady-state levels of AMBRA1 phosphorylation for (A) different levels of AMPK* and (B) different levels of rapamycin*. The dotted curves indicate lower and upper bounds of stable limit cycles. The red curves indicate periods of oscillation (see right vertical axes). In the left panel, the level of rapamycin* is held fixed at zero. In the right panel, the level of AMPK* is held fixed at 30,000 copies per cell. For both panels, the parameters considered in Table 1 are held fixed at their nominal values. The labels SNIC_1_ and SNIC_2_ indicate saddle-node-on-invariant-circle bifurcation points, and the label SuperH indicates a supercritical Hopf bifurcation point. The labels SubH and CF refer to subcritical Hopf and cyclic fold bifurcation points, which are very close to each other (panel A).

To further investigate this behavior, we performed a bifurcation analysis (Fig. 5). We found stable steady states and stable limit cycles of the system at different levels of AMPK* (Fig. 5A) and at different levels of rapamycin* (Fig. 5B). Our results, interpreted using bifurcation theory [25], reveal the qualitative behavior of the system.

The bifurcation diagrams of Fig. 5 reveal that oscillations in AMBRA1 phosphorylation, and alternating phases of autophagy and translation (Fig. 4D–4F), arise through a saddle-node-on-invariant-circle (SNIC) bifurcation as either AMPK* or rapamycin* increases from a low to high level. The period of oscillations is infinite at the bifurcation point and then decreases (Fig. 5A, right vertical axis). The decrease in period is almost entirely caused by a decrease in the duration of the phase where AMBRA1 phosphorylation is low (Fig. B in S2 File). Thus, when the system is oscillating, the duration of the autophagy phase, marked by high AMBRA1 phosphorylation, is insensitive to the level of AMPK*, whereas the duration of the translation phase, marked by low AMBRA1 phosphorylation, decreases with increasing starvation (i.e., increasing AMPK* level). As AMPK* increases, oscillations cease at a cyclic fold (CF) bifurcation point (Fig. 5A). (A cyclic fold bifurcation is also known as a fold bifurcation of limit cycles.) At high AMPK* levels, there is monostability. As AMPK* decreases from a high level, the monostable steady state loses stability through a subcritical Hopf (SubH) bifurcation (Fig. 5A). In the small interval between the SubH and CF bifurcations, a stable steady state and a stable limit cycle coexist. At high rapamycin* levels, there is a monostable steady state (Fig. 5B). As rapamycin* decreases from a high level, the monostable steady state loses stability through a supercritical Hopf (SuperH) bifurcation, giving rise to small-amplitude oscillations that increase in both amplitude and period as the level of rapamycin* decreases (Fig. 5B). As for regulation by AMPK*, where there are oscillations, the period increases as the duration of the translation phase increases, and the duration of the autophagy phase is nearly constant (Fig. B in S2 File).

In summary, Fig. 5A indicates that the system is regulated by AMPK* level such that the system either operates in one of two distinct states or oscillates between these states. Fig. 5B indicates that the system is regulated similarly by rapamycin*, except where there are small-amplitude oscillations. In this regime, there is not a strong cellular commitment to an autophagy state that is distinct from the translation state (or vice versa), which is illustrated in Fig. 4E. The oscillatory behavior of the system can be understood as arising from bistability in the absence (or rather, the apparent or effective absence) of negative feedback from ULK1 to AMPK (Fig. 3). Because negative feedback is relatively slow compared to other processes for nominal parameter values (i.e., the parameter values of Table 1), bistability allows switch-like, all-or-none responses to perturbations on time scales that are fast compared to the time scale of negative feedback. As suggested by the analysis of Fig. 5, a switch from an autophagy state to a translation state (or vice versa) can be reversed by engagement (or extinction) of negative feedback from ULK1 to AMPK. Thus, at least for the nominal parameter values, oscillations arise from a combination of bistable switching on a fast time scale and negative feedback on a slow time scale. These mechanisms allow the system to behave as a relaxation oscillator.

### Sensitivity analysis

**Fig 6:**
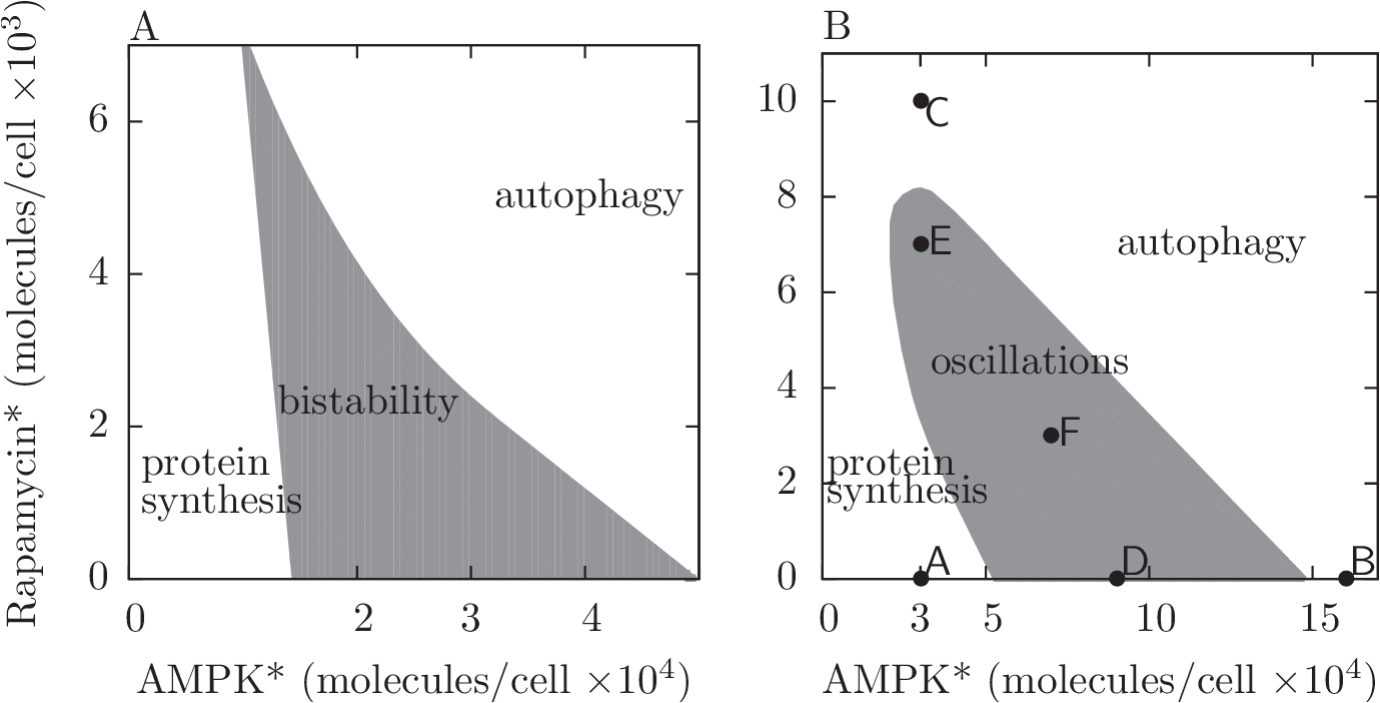
Two-dimensional bifurcation diagrams. (A) The qualitative behavior of the system without negative feedback from ULK1 to AMPK as a function of AMPK* and rapamycin* levels. The shaded region indicates where the system exhibits bistability. (B) The qualitative behavior of the system with negative feedback from ULK1 to AMPK as a function of AMPK* and rapamycin* levels. The shaded region indicates where the system exhibits oscillations. Labeled points correspond to the stress inputs considered in Fig. 4 (e.g., the point labeled F corresponds to the case of Fig. 4F). For both panels, parameter values considered in Table 1 are held fixed at their nominal values.

The full range of possible qualitative behaviors of the system over input space, with and without negative feedback from ULK1 to AMPK, is revealed in Fig. 6 (for the parameter values of Table 1). The two-dimensional bifurcation diagram of Fig. 6A shows the region of bistability in the parameter space of system inputs (the levels of AMPK* and rapamycin*) for the case of no negative feedback from ULK1 to AMPK. Similarly, the two-dimensional bifurcation diagram of Fig. 6B shows the region of oscillatory behavior in the parameter space of inputs for the version of the model with negative feedback. Each labeled point in the parameter space of Fig. 6B corresponds to a time course shown in Fig. 4. Fig. 6 comprehensively characterizes system responses to inputs for a particular parameterization of our model, but of course, the input-output behavior of the system depends on all model parameters. Thus, we investigated the dependence of system behavior on parameter variations away from the parameter values of Table 1.

**Fig 7:**
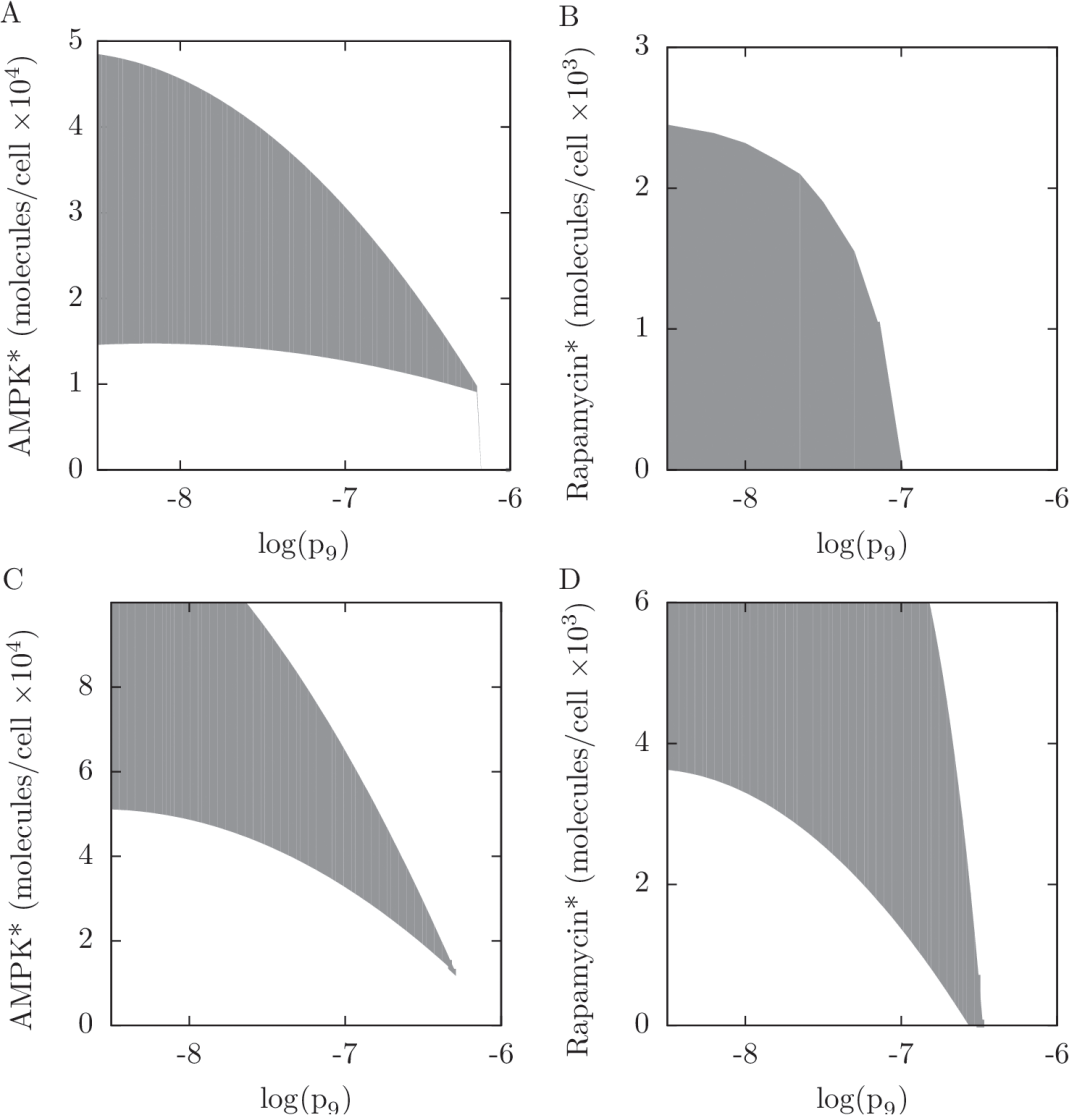
Influence of negative regulation of MTORC1 by AMPK on qualitative system behavior. The rate constant for inhibitory phosphorylation of RPTOR at S792 by AMPK is *p*_9_. The panels shown here are two-dimensional bifurcation diagrams. In the left panels, *p*_9_ and AMPK* level are the bifurcation parameters. In the right panels, *p*_9_ and rapamycin* level are the bifurcation parameters. In the top panels, we consider the system without negative feedback from ULK1 to AMPK. The shaded regions in these panels indicate where the system exhibits bistability. In the bottom panels, we consider the system with negative feedback from ULK1 to AMPK. The shaded regions in these panels indicate where the system exhibits oscillations. Note that the horizontal axes are logarithmic (base 10).

In a preliminary analysis, which was focused on setting parameter values (Materials and Methods), we found that negative regulation of MTORC1 by AMPK [11,26], which is represented by the dashed arrow in Fig. 1, reduces the region of parameter space in which bistability exists (for the special condition of no negative feedback from ULK1 to AMPK). Thus, for the purpose of amplifying the potential effects of mutual inhibition of MTORC1 and ULK1, in the analyses presented above, we set the parameter *p*_9_ to zero (Table 1). In our model, this parameter, a phosphorylation rate constant, characterizes the strength of negative regulation of MTORC1 by AMPK. Here, we investigate the effect of non-zero *p*_9_ on the qualitative behavior of the system through a bifurcation analysis (Fig. 7).

In the two-dimensional bifurcation diagrams of Fig. 7, the bifurcation parameters are *p*_9_ and AMPK* level (Fig. 7A and 7C) and *p*_9_ and rapamycin* level (Fig. 7B and 7D). In Fig. 7A and 7B, as in the analysis of Fig. 3, we consider the special case where negative feedback from ULK1 to AMPK is absent. In the diagrams of Fig. 7A and 7B, the shaded areas mark the regions of parameter space where there is bistability. In Fig. 7C and 7D, we consider the system with negative feedback from ULK1 to AMPK, and the shaded area in each diagram marks the region of parameter space where there is a stable limit cycle. As can be seen, the range of bistability/oscillatory behavior shrinks with increasing strength of negative regulation of ULK1 by AMPK (i.e., with increasing *p*_9_). For *p*_9_ above a threshold value, the system is monostable for all stress inputs and its responses to stimuli are graded. Thus, remarkably, negative regulation of MTORC1 by AMPK, which may require localization of AMPK to the Ragulator complex on lysosomes [27], can determine whether the system makes graded or switch-like responses to inputs. With sufficiently weak negative regulation, as in the analysis of Fig. 5, the system, for the most part, commits to either autophagy or translation, or alternating periods of autophagy and translation. In contrast, with sufficiently strong negative regulation, changes in AMPK* or rapamycin* level elicit gradual changes in autophagy and translation.

**Fig 8:**
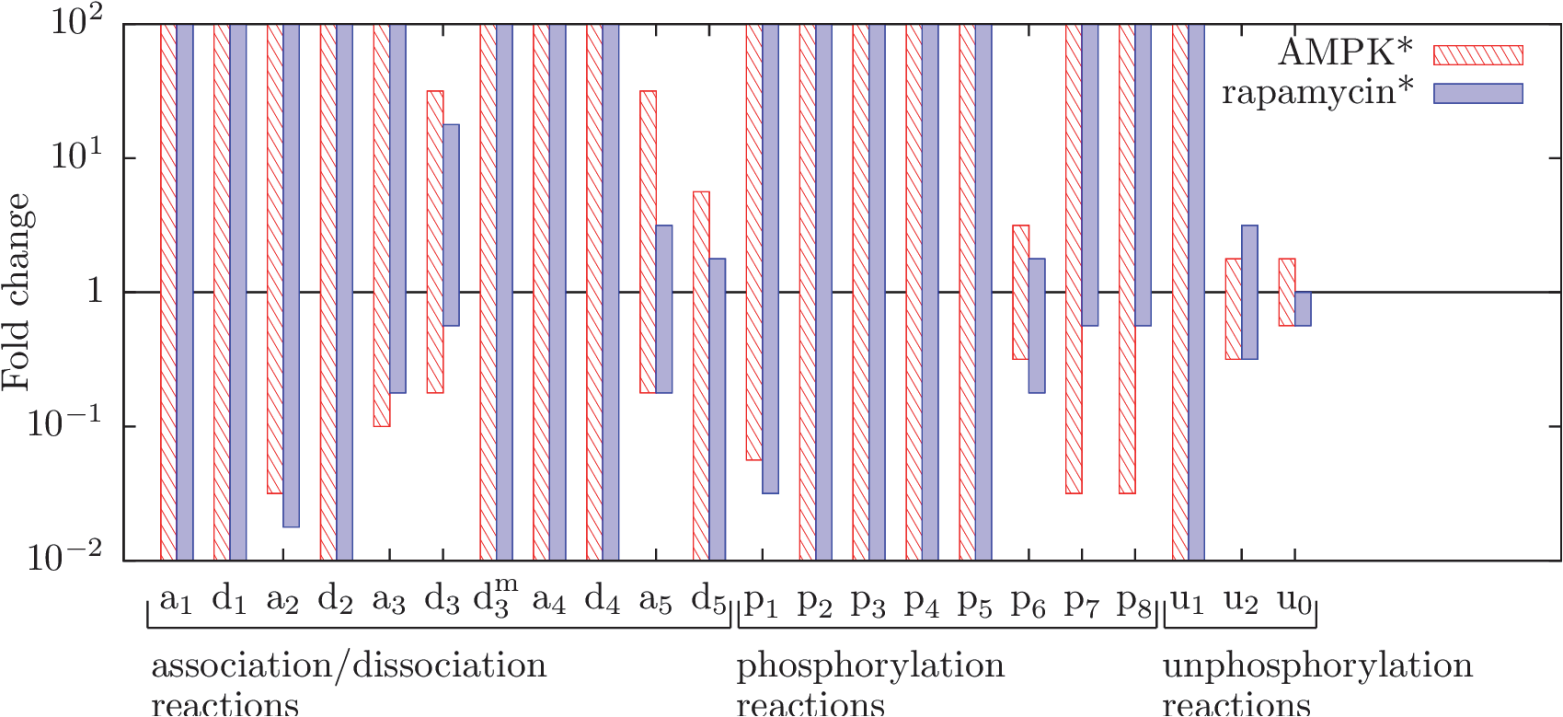
Parameter sensitivity analysis. Each bar corresponds to a rate constant in the model and indicates a range of values for that rate constant over which the following pattern of qualitative behavior is obtained as a stress input (AMPK* or rapamycin* level) is varied: operation in a translation state at low stresses, oscillations between translation and autophagy states at intermediate stresses, and operation in an autophagy state at high stresses. For red bars, the stress input is AMPK* level; we considered levels of AMPK* from 0 to 10^6^ copies per cell. For blue bars, the stress input is rapamycin* level; we considered levels of rapamycin* from 0 to 10^5^ copies per cell. To find the upper and lower bounds of a bar, we varied (in discrete steps) the value of its corresponding parameter individually100-fold above and 100-fold below the parameter’s nominal value, which corresponds to 1 on the vertical axis. For each parameter value tested, scans of AMPK* and rapamycin* levels were performed to determine whether responses to varying levels of stress follow the same pattern as the system with nominal parameter values. The height of a bar serves as a measure of robustness. We considered all rate constants of the model with the exception of *p*_9_. (Recall that the influence of *p*_9_ on system behavior has already been considered, in Fig. 7.)

To further characterize how system behavior depends on parameter values, we systematically varied single parameter values with the goal of delimiting the region of parameter space where patterns of responses to inputs match those of the model with nominal parameter values (i.e., the parameter values of Table 1). We also sought to identify the most sensitive parameters. The results are summarized in Fig. 8. The sensitivity analysis of Fig. 8 was performed by varying each of 22 parameters (all rate constants) alone, with other parameters set at their nominal values. The range of variation for each parameter was 100-fold below and 100-fold above the nominal parameter value. The behavior of the system over the range of variation was determined through simulations (see Fig. C in S2 File and Materials and Methods). We checked the simulation results for a characteristic pattern of responses to increasing levels of the two system inputs, AMPK* and rapamycin*. We considered levels of AMPK* from 0 to 10^6^ copies per cell (under the condition where rapamycin is absent) and levels of rapamycin* from 0 to 10^5^ copies per cell (under the condition where the level of AMPK* is 3×10^4^ copies per cell). The bars in Fig. 8 indicate parameter values where the following pattern of responses was observed: a predominant translation state at low input levels, oscillation between a translation state and an autophagy state at intermediate input levels, and a predominant autophagy state at high input levels (Panel A of Fig. C in S2 File).

Fig. 8 indicates that qualitative system behavior is robust. Out of the 22 parameters considered in our sensitivity analysis, we were able to vary 11 over the entire range of variation considered without observing a change in the characteristic stress response pattern (translation at low stresses, oscillations at intermediate stresses, and autophagy at high stresses). Of course, we did observe changes in quantitative behavior, such as shifts in the range of AMPK* levels where autophagy is predominant.

The two most sensitive parameters, as measured by the lengths of bars in Fig. 8, are *u*_0_ and *u*_2_, which are dephosphorylation rate constants. In the model, as a simplification, we parameterized all dephosphorylation reactions, except two, by the rate constant *u*_0_ (Materials and Methods). As a consequence, the value of *u*_0_ sets the system’s characteristic response time (1/*u*_0_ = 100 s), the time scale on which responses to stress inputs occur. Thus, it is not surprising that system behavior depends most sensitively on the value of *u*_0_. The rate constant *u*_2_ characterizes dephosphorylation of ULK1-phosphorylated sites in AMPK. These sites are involved in negative feedback from ULK1 to AMPK. Thus, the value of *u*_2_ controls the time scale of negative feedback, which as we have seen above, plays an important role in determining qualitative system behavior. The third most sensitive parameter is *p*_6_ (Fig. 8), the phosphorylation rate constant that characterizes ULK1-mediated phosphorylation of AMPK. Thus, this parameter is influential for the same reason that *u*_2_ is influential.

## Discussion

Our modeling study suggests that the AMPK-MTORC1-ULK1 regulatory network, which is represented diagrammatically in Figs. 1 and 2, has the potential to respond nonlinearly to stress inputs, largely as a consequence of mutual inhibition of MTORC1 and ULK1, which provides positive feedback. Slow negative feedback from ULK1 to AMPK also plays an important role in generating nonlinear responses. For the parameter values of Table 1, we expect the network to respond to a moderate stress input by oscillating between periods of autophagy and translation (Figs. 4–6). The amplitude of these oscillations, especially for a stress that manifests as a change in the intrinsic kinase activity of AMPK (e.g., a change in the cellular AMP:ATP ratio), is expected to be fairly insensitive to the stress level (Fig. 5). The duration of the autophagy phase is also expected to be insensitive to stress level, but in contrast, the relatively long duration of the translation phase is expected to decrease with increasing stress level (Fig. B in S2 File). Except for special conditions that give rise to small-amplitude oscillations, such as the condition considered in Fig. 4E, the processes of autophagy and translation are expected to be activated in a mutually exclusive manner. Furthermore, the operating states of the network that promote autophagy and translation are predicted to be characterized by distinct patterns of site-specific protein phosphorylation (Fig. A in S2 File). In response to a severe stress, we expect the network to exhibit a monostable steady state where autophagy is activated and translation is repressed, as illustrated in Fig. 4B and 4C. The opposite is expected in the absence of stress, as illustrated in Fig. 4A. These expected qualitative behaviors, which could potentially be detected experimentally, are robust to parameter uncertainty (Fig. 8).

Despite robustness, behavior is malleable and there are epistemic uncertainties about model structure. If negative feedback from ULK1 to AMPK is ablated, oscillatory behavior is no longer expected. Instead, the network is expected to exhibit bistability (Fig. 3), which is characterized by all-or-none, switch-like responses and hysteresis (i.e., history dependence). For example, without negative feedback, the network may not be able to shut off autophagy once autophagy has been activated by rapamycin treatment, even after complete clearance of rapamycin (Fig. 3, right panels). This dysfunctional behavior is avoided with negative feedback from ULK1 to AMPK (compare the top right panel of Fig. 3 with the right panel of Fig. 5), which is consistent with the view that negative feedback protects against excessive autophagy [28]. Network behavior can also be changed qualitatively by modifying the strength of negative regulation of RPTOR (and MTORC1) by AMPK. If negative regulation of RPTOR by AMPK is strong (vs. zero, or sufficiently weak), the network may exhibit graded responses to stress inputs (Fig. 7). These results suggest that perturbations affecting negative feedback of ULK1 to AMPK and/or negative regulation of RPTOR by AMPK could potentially have profound effects on network behavior. These perturbations could include somatic mutations that alter the substrates of ULK1 in AMPK that are involved in negative feedback or the substrates of AMPK in RPTOR, as well as dietary or therapeutic compounds and cell-to-cell variations in protein abundances that affect the relevant kinase and phosphatase activities.

It should be noted that our model does not consider the effects of long-term regulation of autophagy induction and translation, including regulation at the level of gene expression. For instance, we do not consider transcription factor EB (TFEB), which promotes lysosome biogenesis and autophagy by increasing the transcription of genes underlying these processes [29]. TFEB is directly bound by MTORC1 on lysosomes, restricting it to the cytoplasm [30]. Upon nutrient starvation, TFEB is released from MTORC1, permitting its nuclear translocation and activity. Similarly, we do not consider ZKSCAN3, a transcription factor inhibited during nutrient starvation that represses many autophagy and lysosome genes [31]. Clearly, regulation at the level of transcription is important, as are many other factors, such as lysosome trafficking and the subcellular locations of AMPK, MTORC1 and ULK1. Our view is that a basic understanding of the AMPK-MTORC1-ULK1 network is a prerequisite for understanding these additional layers of regulation.

The range of qualitative behaviors that can potentially be exhibited by the AMPK-MTORC1-ULK1 regulatory network is consistent with results from earlier theoretical/modeling studies focused on understanding the design principles of simple regulatory circuits and the behaviors of various specific systems. For example, it is well known from such studies that relaxation oscillations can arise from a hysteretic switch (i.e., bistability) [32]. We found that the main features of the AMPK-MTORC1-ULK1 network that permit the system to behave as a relaxation oscillator are 1) mutual inhibition of MTORC1 and ULK1, which involves nonlinear, multi-site phosphorylation of RPTOR by ULK1, and 2) slow negative feedback from ULK1 to AMPK. The first feature allows the system to behave as a hysteric switch on short time scales (Fig. 3). In initial exploratory simulations, we found that multi-site phosphorylation of RPTOR by ULK1 provides the nonlinearity needed for mutual inhibition to give rise to hysteretic switching. This potential contribution of multi-site phosphorylation to hysteretic switching has been characterized in earlier work focused on cell-cycle control [33,34]. The second feature gives rise to oscillatory behavior (Fig. 5).

Recently, Lomnitz and Savageau [35] used the method of mathematically controlled comparison to characterize the ability of various regulatory circuits to produce oscillations. They found that a nested positive feedback circuit design (i.e., a circuit in which a fast positive feedback loop is nested within a slow negative feedback loop) more robustly generates oscillations than a nested negative-positive feedback circuit design. This finding is consistent with our observation that negative regulation of RPTOR by AMPK promotes graded (vs. oscillatory) responses to stress inputs (Fig. 7). Without negative regulation of RPTOR by AMPK, the AMPK-MTORC1-ULK1 network has the topology of a nested positive feedback circuit (taking mutual inhibition or double negative feedback to be equivalent to positive feedback) and, as expected, appears to be prone to exhibit oscillations. In contrast, with negative regulation of RPTOR by AMPK, the positive (double negative) feedback is attenuated, which makes the network less prone to exhibit oscillations.

Is there any evidence that the AMPK-MTORC1-ULK1 network behaves as predicted? There is some experimental evidence consistent with hysteretic switching and/or oscillations arising in this network. Procaccini et al. [36] inferred that oscillations in MTOR activity play a role in setting the responsiveness of regulatory T cells, but these oscillations were not characterized, nor was their source elucidated. More recently, Xu et al. [37] observed that phosphorylation of ribosomal protein S6 (RPS6), a downstream target of MTORC1, is regulated in a switch-like manner in response to rapamycin treatment, with a bimodal distribution of RPS6 phosphorylation among cells at an intermediate rapamycin dose but a unimodal distribution at either a low or high dose. This behavior is suggestive, but not diagnostic, of hysteretic switching. In contrast, autophagy, as assayed by counting MAP1LC3A-positive autophagic vesicles, increased in a graded fashion in response to increasing doses of rapamycin [37]. These results may be consistent with the behavior predicted by Fig. 5B if oscillations are asynchronous (across different cells in a population and/or across different locations of autophagosome production within a single cell), given that the translation phase during oscillations is longer than the autophagy phase and that the translation phase becomes shorter with increasing rapamycin dose (Panel B of Fig. B in S2 File). In any case, the readouts measured by Xu et al. [37] lie outside the scope of our model. Direct tests of model predictions would require specific measurements, such as real-time single-cell measurements of kinase activity enabled by a Förster resonance energy transfer (FRET)-based biosensor (to observe oscillations) or high-throughput unbiased assays of site-specific phosphorylation enabled by quantitative mass spectrometry (MS)-based proteomics (to observe the distinct patterns of phosphorylation predicted for autophagy and translation states).

Above, we raised the possibility that there may be localized, asynchronous oscillations across different sites of autophagosome production (and protein synthesis) in a cell. We raised this possibility because of the punctate distribution of ULK1 [38,39,40] and other evidence indicating that subcellular compartmentalization is an important aspect of both protein synthesis and autophagy [41]. Because the processes captured in our model have a time scale of minutes to hours, we would expect diffusion, which is relatively fast, to synchronize oscillations across the relevant subcellular compartments of a cell. However, we caution that aleatory variability (e.g., fluctuations arising from the stochastic nature of chemical reactions) could potentially mask oscillations at the single-cell level by giving rise to asynchrony across the subcellular compartments where AMPK, MTORC1, and ULK1 are locally interacting if these molecular players are not free to mix, which could be the case if their localization is regulated. We fully expect that heterogeneity in protein copy numbers (i.e., heterogeneity in the numbers of protein copies per cell), which is a well-characterized general feature of cell populations [42,43,44], would mask oscillations at the population level. Thus, experiments aimed at detecting oscillations should include single-cell readouts, at least. Readouts with fine spatial resolution (e.g., organelle-level readouts) may be required.

Returning to the issue of epistemic uncertainty, we caution that the structure of our model is based on simplifying assumptions that may need to be revisited as new data emerges. The model omits several interactions and sites of phosphorylation that have been reported in the literature. As discussed in Materials and Methods, most of these omitted sites and interactions are seemingly redundant, meaning that they appear to play a role that would strengthen influences and effects already considered in the model. However, others are enigmatic, or beyond the intended scope of our study, which is limited to direct interactions among AMPK, MTORC1 and ULK1 and the noted inputs and outputs. Thus, the model does not include, for example, TRAF6-mediated positive feedback from AMBRA1 to ULK1 [38], indirect regulation of MTORC1 by AMPK via TSC1/TSC2/RHEB [45], MTORC1-mediated phosphorylation of components of the ULK1 complex (e.g., ATG13) other than ULK1 [14,23], the Ragulator complex or Rag GTPases [46], or association of MTOR with RICTOR in MTORC2 [47]. Finally, it should be noted that the model does not account for isoform-specific effects, which in general are poorly understood. We note that there are multiple isoforms of ULK1 [48] and multiple isoforms of each of the three subunits of AMPK [49].

Negative feedback regulation from ULK1 to AMPK can be viewed as a regulatory mechanism that protects a cell against excessive autophagy (e.g., an overshooting response to a stress input), which could compromise its viability. It is intriguing to consider the possibility that negative feedback regulation, in combination with mutual inhibition of MTORC1 and ULK1, may have the more sophisticated function of generating pulses of autophagy separated by periods of translation that allow use of the amino acids generated through autophagy and recovery from self-eating. Dunlop and Tee [50] hypothesized that alternating periods of autophagy and translation may be important for homeostasis, to avoid the detrimental effects of activating either MTORC1 or ULK1 for too long a period. Based on the results presented here, oscillations between autophagy and translation states appear to be entirely feasible. Our model connects regulatory influences to underlying molecular mechanisms and the results of analyzing the model suggest numerous non-obvious testable predictions, which will be useful in future experimental investigations of the behavior of the AMPK-MTORC1-ULK1 regulatory network.

## Materials and Methods

### Modeling approach

To formulate the model, we used a rule-based approach, which simplifies the consideration of site-specific details (e.g., tracking the phosphorylation status of amino acid residues) [51]. We used the model-specification language of BioNetGen, which is called BNGL, to define the types of molecules included in our model and to write rules for interactions. The conventions of BNGL are described elsewhere in detail [52,53]. A rule provides necessary and sufficient conditions for occurrence of an interaction and a rate law that governs all reactions implied by the rule. The model accounts for seven types of molecules: (FKBP1A-bound) rapamycin, a natural product, and the proteins AMPK, MTOR, RPTOR, ULK1, EIF4EBP1, and AMBRA1. The model includes 27 unidirectional rules and 2 bidirectional rules. Below, we present and discuss the BNGL-encoded molecule type definitions and rules that comprise the model.

### Molecule type definitions

The focus of this section is on explaining the formal representation of molecules considered in the model.

#### Rapamycin*

Rapamycin in complex with FKBP1A (UniProt entry P62942) binds MTOR [17,18], which prevents MTOR from associating with RPTOR [54]. For simplicity, we only consider the form of rapamycin in complex with FKBP1A, which we denote as rapamycin*. In the model, this form of rapamycin is named rapa and is taken to have a single component named mtor, which is responsible for interaction with MTOR:

rapa(mtor)

The level of rapamycin* is treated as an input. The level of rapamycin* is not affected by the dynamics captured in the model. We chose to study responses to rapamycin rather than physiological stimuli, such as amino acids or growth factors, because rapamycin treatment is a stimulus that can be readily manipulated in the laboratory and rapamycin directly modulates MTORC1 activity. Regulation of MTORC1 activity in response to physiological stimuli is less direct and mediated by a complex regulatory network, which integrates multiple signals.

#### AMPK*

As a simplification, we only consider the form of AMPK with activating phosphorylation in its kinase domain, i.e., the form of AMPK with phosphorylation at T172 (T183) in the PRKAA2 (PRKAA1) isoform of the α subunit of AMPK (UniProt entries P54646 and Q13131). We denote this form of AMPK as AMPK*. In the model, AMPK* interacts directly with ULK1 via an undefined region in AMPK*. Furthermore, AMPK* phosphorylates sites in RPTOR and ULK1, and AMPK* is a substrate of ULK1. The kinase domain of AMPK* is considered implicitly. We represent AMPK* as follows:

AMPK(ulk1,ST∼0∼P)

The ulk1 component represents the undefined region in AMPK* that mediates interaction with ULK1. The ST component represents serine/threonine residues in the α subunit of AMPK that are phosphorylated by ULK1. This component is taken to have an internal state, which is either “unphosphorylated” (0) or “phosphorylated” (P). Internal states are abstractions useful for representing local properties of sites, such as location, conformation, or post-translational modification status. In a molecule type definition, the names of all possible internal states of a site are listed after the name of that site, with each state name being prefixed by a tilde. The level of AMPK* is treated as an input. The level of AMPK* is not affected by the dynamics captured in the model.

#### MTOR

MTOR (UniProt entry P42345) interacts directly with rapamycin* and RPTOR [54]. The interactions with rapamycin* and RPTOR, which are mutually exclusive, are mediated by the FRB domain and HEAT repeats in MTOR, respectively [17,18,54]. Furthermore, MTOR, when part of MTORC1, phosphorylates substrates in ULK1 [10,14,55] and EIF4EBP1 [56,57]. We consider the kinase domain of MTOR implicitly. Thus, MTOR is represented as follows:

MTOR(HEAT,FRB)

In the model, MTOR’s kinase is taken to be active when bound to RPTOR, such that only MTOR in complex with RPTOR is able to phosphorylate substrates. As a simplification, the only components of MTORC1 that we track in our model are MTOR and RPTOR. Thus, the complex of MTOR and RPTOR is taken to be equivalent to MTORC1.

#### RPTOR

RPTOR (UniProt entry Q8N122) interacts directly with MTOR, ULK1, and EIF4EBP1. The interactions with MTOR and EIF4EBP1 are mediated by WD40 repeats and the RNC domain in RPTOR, respectively. The interaction with ULK1 is mediated by an undefined region in RPTOR. Several sites in RPTOR, including S792, S855, and S859, are substrates of AMPK and/or ULK1 [11,15]. We represent RPTOR as follows:

RPTOR(RNC,ulk1,WD40,S792∼0∼P,S855_S859∼0∼P∼PP)

The ulk1 component represents the undefined region in RPTOR that mediates interaction with ULK1. The S792 component is allowed to have one of two internal states: 0, which is taken to represent the unphosphorylated form of S792, or P, which is taken to represent the phosphorylated form of S792. As a simplification, the S855 and S859 residues are lumped together and represented by a single component named S855_S859. This component is taken to have one of three internal states: unphosphorylated (0), singly phosphorylated (P), or doubly phosphorylated (PP).

#### ULK1

ULK1 (UniProt entry O75385) interacts directly with RPTOR and AMPK; these interactions are mediated by undefined regions within the proline/serine-rich (PS) domain of ULK1 [10]. Several sites in ULK1, including S317, S758, and S778, are substrates of MTORC1 or AMPK, and ULK1 phosphorylates several substrates in RPTOR, AMPK and AMBRA1. We consider the kinase domain of ULK1 implicitly. Thus, ULK1 is represented as follows:

ULK1(straptor,stampk,S317∼0∼P,S758∼0∼P,S778∼0∼P)

The components named straptor and stampk represent the undefined regions responsible for interactions with RPTOR and AMPK. The component S758 is a substrate of MTORC1, and the components S317 and S778 are substrates of AMPK. These components are each taken to have one of two internal states: unphosphorylated (0) or phosphorylated (P). In the model, ULK1’s kinase is taken to be active when phosphorylated at both S317 and S778 and not phosphorylated at S758.

#### EIF4EBP1

When recruited to RPTOR via its RCR domain, EIF4EBP1 (UniProt entry Q13541) can be phosphorylated by MTORC1 at several sites, including T37, T46, S65 and T70 [58]. Phosphorylation of S65 and T70 is more sensitive to rapamycin treatment than T37 and T46 [59,60]. Thus, we represent EIF4EBP1 as follows:

EIF4EBP1(RCR,S65_T70∼0∼P)

As can be seen, as a simplification, we represent S65 and T70 (and other less rapamycin sensitive sites) together by a single component named S65_T70. This component is taken to have an internal state: either unphosphorylated (0) or phosphorylated (P). EIF4EBP1 is included in the model as a representative of MTORC1 substrates involved in regulating translation. EIF4EBP1 represses translation when hypophosphorylated and is inert when phosphorylated by MTORC1.

#### AMBRA1

AMBRA1 (UniProt entry Q9C0C7) is phosphorylated by ULK1. We represent AMBRA1 as follows:

AMBRA1(ST∼0∼P)

The ST component represents serine/threonine residues that are phosphorylated by ULK1. This component is taken to have an internal state: either unphosphorylated (0) or phosphorylated (P). AMBRA1 is included in the model as a reporter of ULK1 kinase activity and autophagy level. Phosphorylation of AMBRA1 by ULK1 promotes autophagy.

### Rules

The focus of this section is on explaining the formal representation of interactions considered in the model. The interactions discussed here are those that seemed most relevant for understanding mutual inhibition of MTORC1 and ULK1 after a literature search aimed at identifying interactions among AMPK, MTORC1, and ULK1. Not all known interactions among this triad are included in the model; omitted interactions are considered below and also in the Discussion section.

#### Rapamycin* binds MTOR

FKBP1A-bound rapamycin (rapamycin*) can interact with MTOR provided that MTOR is not bound to RPTOR. Rapamycin* binding to MTOR and RPTOR binding to MTOR are mutually exclusive. We represent reversible binding of rapamycin* to MTOR as follows:

rapa(mtor)+MTOR(HEAT,FRB)<->rapa(mtor!1).MTOR(HEAT,FRB!1)a1,d1 (1)

where a1 and d1 are the forward and reverse rate constants for this interaction. Note that the rule of Equation (1) has both a forward and reverse direction, as indicated by the symbol “<->.” Thus, the rule can be read from left to right, or right to left; it is one of the two reversible rules included in the model. As indicated by sharing of the bond index “1” by the mtor and FRB components on the right-hand side of the rule in Equation (1), rapamycin* binds the FRB domain in MTOR. (Bond indices are prefixed by an exclamation mark.) Inclusion of the HEAT component in the left-hand side of Equation (1) without specification of a binding partner indicates that this component must be free in order for the rule to be applicable. This constraint is introduced to ensure that rapamycin* and RPTOR bind MTOR with mutual exclusivity. In the model, only RPTOR interacts with HEAT repeats in MTOR. Thus, the rule of Equation (1) indicates that MTOR must be free of RPTOR to interact with rapamycin*. We note that the reverse (i.e., right-to-left) reading of the rule of Equation (1) indicates that the HEAT repeats in MTOR must be free in order for rapamycin* to dissociate from MTOR. Writing the rule with this restriction, which is spurious (but inconsequential for the model as specified), has the benefit of allowing for concise specification of the forward and reverse rules for binding of rapamycin* to MTOR. Only a single line of BNGL code is necessary. The restriction has no consequence whatsoever because the rules of the model never allow the HEAT and FRB sites in MTOR to be bound at the same time. If the model were modified in some way to allow simultaneous binding of these sites, then the rule of Equation (1) would likely need to be rewritten as two separate, unidirectional rules.

#### RPTOR binds MTOR

RPTOR can interact with MTOR provided that MTOR is not bound to rapamycin*. We represent reversible binding of RPTOR to MTOR as follows:

RPTOR(WD40)+MTOR(HEAT,FRB)<->RPTOR(WD40!1).MTOR(HEAT!1,FRB)a2,d2 (2)

where a2 and d2 are the forward and reverse rate constants for this interaction. As indicated, WD40 repeats in RPTOR interact with HEAT repeats in MTOR. Inclusion of the FRB component in the left-hand side of Equation (2) without specification of a binding partner indicates that this component must be free in order for the rule to be applicable. In the model, only rapamycin* interacts with FRB domain in MTOR. Thus, the rule of Equation (2) indicates that MTOR must be free of rapamycin* in order to interact with RPTOR.

#### RPTOR binds ULK1

RPTOR can interact reversibly with ULK1. This interaction, which is mediated by undefined regions in RPTOR and ULK1, is destabilized by phosphorylation of RPTOR at S792, S855, and S859. We represent reversible binding of RPTOR to ULK1 using three unidirectional rules, as follows:

$$\begin{array}{l} \text{RPTOR}\left(\text{RNC,ulk1,S792\textasciitilde{}0,S855\_Ser859\textasciitilde{}0}\right)\text{+ULK1}\left(\text{straptor}\right)\text{-> \textbackslash{} }\\ \text{RPTOR}\left(\text{RNC,ulk1!1,S792\textasciitilde{}0,S855\_Ser859\textasciitilde{}0}\right)\text{.ULK1}\left(\text{straptor!1}\right)\;\text{a3} \end{array}$$ (3A)

RPTOR(ulk1!1).ULK1(straptor!1)->RPTOR(ulk1)+ULK1(straptor)d3 (3B)

$$\begin{array}{l} \text{RPTOR}\left(\text{ulk1!1,S792\textasciitilde{}P,S855\_Ser859\textasciitilde{}PP}\right)\text{.ULK1}\left(\text{straptor!1}\right)\text{-> \textbackslash{} }\\ \text{ RPTOR}\left(\text{ulk1,S792\textasciitilde{}P,S855-9\textasciitilde{}PP}\right)\text{+ULK1}\left(\text{straptor}\right)\;\text{d3max} \end{array}$$ (3C)

Note that unidirectionality is indicated by the symbol “->.” A unidirectional rule is only read from left to right. The first rule, Equation (3A), indicates that RPTOR, when free of EIF4EBP1 (which binds the RNC domain in RPTOR) and not phosphorylated at S792, S855, and S859, is able to associate with ULK1 with rate constant a3. (This rule requires that the cognate binding sites in RPTOR and ULK1, which are represented by the components named ulk1 and straptor, be free.) The second rule, Equation (3B), indicates that RPTOR is able to dissociate from ULK1 with rate constant d3. Dissociation can occur whenever a bond between RPTOR and ULK1 exists. In other words, according to this rule, there are no contextual constraints on dissociation. The third rule, Equation (3C), indicates that dissociation occurs with a different rate constant, d3max (>d3), when the indicated sites in RPTOR are phosphorylated. We note that a backslash (\) is used to mark a line break in BNGL.

#### RPTOR binds EIF4EBP1

RPTOR can interact reversibly with EIF4EBP1. This interaction occurs between the RNC domain in RPTOR and the RCR domain in EIF4EBP1. We represent reversible binding of RPTOR to EIF4EBP1 using two unidirectional rules, as follows:

$$\begin{array}{l} \text{RPTOR}\left(\text{RNC,ulk1,S792\textasciitilde{}0,S855\_Ser859\textasciitilde{}0}\right)\text{+EIF4EBP1}\left(\text{RCR,S65\_T70\textasciitilde{}0}\right)\text{-> \textbackslash{} }\\ \text{ RPTOR}\left(\text{RNC!1,ulk1,S792\textasciitilde{}0,S855\_Ser859\textasciitilde{}0}\right)\text{.EIF4EBP1}\left(\text{RCR!1,S65\_T70\textasciitilde{}0}\right)\;\text{a4} \end{array}$$ (4A)

RPTOR(RNC!1).EIF4EBP1(RCR!1)->RPTOR(RNC)+EIF4EBP1(RCR)d4 (4B)

The first rule, Equation (4A), indicates that RPTOR, when free of ULK1 (which interacts with an undefined region in RPTOR that we refer to as ulk1) and not phosphorylated at S792, S855, and S859, is able to associate with EIF4EBP1 with rate constant a4. The sites that mediate association, the RNC domain in RPTOR and the RCR domain in EIF4EBP1, must be free and available for interaction, as indicated. The second rule, Equation (4B), indicates that RPTOR is able to dissociate from EIF4EBP1 with rate constant d4.

#### AMPK binds ULK1

AMPK can interact reversibly with ULK1. This interaction occurs between an undefined region in AMPK, denoted ulk1, and an undefined region in ULK1, denoted stampk. We represent reversible binding of AMPK to ULK1 using two unidirectional rules, as follows:

$$\begin{array}{l} \text{AMPK}\left(\text{ulk1,T172\textasciitilde{}P}\right)\text{+ULK1}\left(\text{stampk,S758\textasciitilde{}0}\right)\text{-> \textbackslash{}}\\ \text{AMPK}\left(\text{ulk1!1,T172\textasciitilde{}P}\right)\text{.ULK1}\left(\text{stampk!1,S758\textasciitilde{}0}\right)\;\text{a5} \end{array}$$ (5A)

AMPK(ulk1!1).ULK1(stampk!1)->AMPK(ulk1)+ULK1(stampk)d5 (5B)

The first rule, Equation (5A), indicates that AMPK, when phosphorylated at T172 and free of ULK1, is able to associate with ULK1, provided that ULK1 is not phosphorylated at S758. Recall that phosphorylation of S758 inhibits AMPK-ULK1 interaction. Association of AMPK and ULK1 occurs with rate constant a5. The sites ulk1 and stampk must be free for association to occur, as indicated. The second rule, Equation (5B), indicates that AMPK is able to dissociate from ULK1 with rate constant d5.

#### MTORC1 phosphorylates ULK1 at S758

We represent MTORC1-mediated inhibitory phosphorylation of ULK1 at S758 using a unidirectional rule, as follows:

$$\begin{array}{l} \text{MTOR}\left(\text{HEAT!1}\right)\text{.RPTOR}\left(\text{WD40!1,ulk1!2}\right)\text{.ULK1}\left(\text{straptor!2,stampk,S758\textasciitilde{}0}\right)\text{-> \textbackslash{}}\\ \text{MTOR}\left(\text{HEAT!1}\right)\text{.RPTOR}\left(\text{WD40!1,ulk1!2}\right)\text{.ULK1}\left(\text{straptor!2,stampk,S758\textasciitilde{}P}\right)\;\text{p1} \end{array}$$ (6)

where p1 is a (pseudo first-order) rate constant. This rule requires that the stampk site in ULK1 be free (of AMPK) in order for MTORC1 to phosphorylate S758. This requirement is introduced for the following reason. It is known that phosphorylation of S758 prevents AMPK from binding ULK1. Thus, we assume that S758 is in the interface between these two proteins. If this assumption is correct, then it follows that S758 should not be accessible for phosphorylation when ULK1 is bound to AMPK (i.e., when the stampk site is bound). The rule of Equation (6) indicates that phosphorylation of S758 by MTORC1 further requires MTOR, RPTOR and ULK1 to be together in a complex. In the model, this complex forms through association of RPTOR with MTOR according to Equation (2) and through association of RPTOR with ULK1 according to Equation (3). The order of association events is of no importance. In the model, components of MTORC1 beyond MTOR and RPTOR (e.g., MLST8) are considered implicitly. We assume that MTORC1 is fully formed and competent to phosphorylate substrates whenever MTOR and RPTOR are in association. Phosphorylation of S758 in ULK1 is inhibitory because phosphorylation of this residue prevents AMPK from binding ULK1 (see Equation (5A)).

#### MTORC1 phosphorylates EIF4EBP1 at S65 and T70

We represent MTORC1-mediated phosphorylation of S65 and T70 in EIF4EBP1 using a unidirectional rule, as follows:

$$\begin{array}{l} \text{MTOR}\left(\text{HEAT!1}\right)\text{.RPTOR}\left(\text{WD40!1,RNC!2}\right)\text{.EIF4EBP1}\left(\text{RCR!2,S65\_T70\textasciitilde{}0}\right)\text{->\textbackslash{}}\\ \text{MTOR}\left(\text{HEAT!1}\right)\text{.RPTOR}\left(\text{WD40!1,RNC!2}\right)\text{.EIF4EBP1}\left(\text{RCR!2,S65\_T70\textasciitilde{}P}\right)\;\text{p2} \end{array}$$ (7)

where p2 is a (pseudo first-order) rate constant. Equation (7) indicates that phosphorylation of S65 and T70 requires MTOR, RPTOR and EIF4EBP1 to be together in a complex. Recall that S65 and T70 in EIF4EBP1 are represented, for simplicity, as a single component named S65_T70. (See the molecule type definition for EIF4EBP1.)

#### ULK1 phosphorylates RPTOR at S792, S855, and S859

We represent ULK1-mediated inhibitory phosphorylation of S792, S855, and S859 in RPTOR using three unidirectional rules, as follows:

$$\begin{array}{l} \text{RPTOR}\left(\text{ulk1!1,S792\textasciitilde{}0}\right)\text{.ULK1}\left(\text{straptor!1,S317\textasciitilde{}P,S778\textasciitilde{}P}\right)\text{-> \textbackslash{}}\\ \text{RPTOR}\left(\text{ulk1!1,S792\textasciitilde{}P}\right)\text{.ULK1}\left(\text{straptor!1,S317\textasciitilde{}P,S778\textasciitilde{}P}\right)\;\text{p3} \end{array}$$ (8A)

$$\begin{array}{l} \text{RPTOR}\left(\text{ulk1!1,S855\_S859\textasciitilde{}0}\right)\text{.ULK1}\left(\text{straptor!1,S317\textasciitilde{}P,S778\textasciitilde{}P}\right)\text{-> \textbackslash{}}\\ \text{RPTOR}\left(\text{ulk1!1,S855\_S859\textasciitilde{}P}\right)\text{.ULK1}(\text{straptor!1,S317\textasciitilde{}P,S778\textasciitilde{}P})\;\text{2*p4} \end{array}$$ (8B)

$$\begin{array}{l} \text{RPTOR}\left(\text{ulk1!1,S855\_S859\textasciitilde{}P}\right)\text{.ULK1}\left(\text{straptor!1,S317\textasciitilde{}P,S778\textasciitilde{}P}\right)\text{-> \textbackslash{}}\\ \text{RPTOR}\left(\text{ulk1!1,S855\_S859\textasciitilde{}PP}\right)\text{.ULK1}\left(\text{straptor!1,S317\textasciitilde{}P,S778\textasciitilde{}P}\right)\;\text{p4} \end{array}$$ (8C)

where p3 and p4 are (pseudo first-order) rate constants. As indicated, phosphorylation of S792, S855, and S859 in RTPOR by ULK1 requires that RPTOR and ULK1 be connected through the binding sites ulk1 in RPTOR and straptor in ULK1. Phosphorylation of S317 and S778 in ULK1, which makes straptor competent for interaction with ulk1 in RPTOR, is an additional prerequisite for ULK1-mediated phosphorylation of S792, S855, and S859 in RPTOR. Recall that S855 and S858 in RPTOR are represented, for simplicity, as a single component named S855_S859, which is allowed to be unphosphorylated (0), singly phosphorylated (P), or doubly phosphorylated (PP). (See the molecule type definition for RPTOR.) The rate constant in the rule of Equation (8B) is specified as twice that for the rule of Equation (8C) because the 0 to P state transition for site S855_S859 (i.e., the transition from an unphosphorylated state to a singly phosphorylated state) is expected to occur twice as fast as the P to PP transition (i.e., the transition from a singly to doubly phosphorylated state).

#### ULK1 phosphorylates AMBRA1 at S/T residues

We represent ULK1-mediated phosphorylation of AMBRA1 at undefined serine and threonine (S/T) residues using a unidirectional rule, as follows:

$$\begin{array}{l} \text{ULK1}\left(\text{straptor,S317\textasciitilde{}P,S778\textasciitilde{}P}\right)\text{+AMBRA1}\left(\text{ST\textasciitilde{}0}\right)\text{-> \textbackslash{}}\\ \text{ULK1}\left(\text{straptor,S317\textasciitilde{}P,S778\textasciitilde{}P}\right)\text{+AMBRA1}\left(\text{ST\textasciitilde{}P}\right)\;\text{p5} \end{array}$$ (9)

where p5 is a (pseudo first-order) rate constant. Equation (9) indicates that ULK1, when phosphorylated at S317 and S778 and not bound to RPTOR (i.e., when the straptor site in ULK1 is free), is able to phosphorylate AMBRA1 at undefined S/T residues, which are represented as a single component named ST. This rule reflects the inhibitory effect of RPTOR on ULK1 kinase activity and the requirement for activating phosphorylation at S317 and S778, which is mediated by AMPK. Note that ULK1 and AMBRA1 are not required to form a complex for ULK1-mediated phosphorylation of AMBRA1 to occur. Thus, we are assuming that the enzymatic reactions defined by the rule of Equation (9) are occurring in the regime of substrate limitation, far from enzyme saturation.

#### ULK1 phosphorylates AMPK at S/T residues

We represent ULK1-mediated phosphorylation of AMPK at undefined serine and threonine residues using a unidirectional rule, as follows:

$$\begin{array}{l} \text{ULK1}\left(\text{straptor,S317\textasciitilde{}P,S778\textasciitilde{}P}\right)\text{+AMPK}\left(\text{ST\textasciitilde{}0}\right)\text{-> \textbackslash{}}\\ \text{ULK1}\left(\text{straptor,S317\textasciitilde{}P,S778\textasciitilde{}P}\right)\text{+AMPK}\left(\text{ST\textasciitilde{}P}\right)\;\text{p6} \end{array}$$ (10)

where p6 is a (pseudo first-order) rate constant. The rule of Equation (10) is the same as that of Equation (9) except that the ULK1 substrates, which are represented by the component named ST, are in AMPK instead of AMBRA1.

#### AMPK phosphorylates ULK1 at S317 and S778

We represent AMPK-mediated activating phosphorylation of ULK1 at S317 and S778 using two unidirectional rules, as follows:

$$\begin{array}{l} \text{AMPK}\left(\text{ulk1!1,T172\textasciitilde{}P,ST\textasciitilde{}0}\right)\text{.ULK1}\left(\text{stampk!1,straptor,S317\textasciitilde{}0}\right)\text{-> \textbackslash{}}\\ \text{AMPK}\left(\text{ulk1!1,T172\textasciitilde{}P,ST\textasciitilde{}0}\right)\text{.ULK1}\left(\text{stampk!1,straptor,S317\textasciitilde{}P}\right)\;\text{p7} \end{array}$$ (11A)

$$\begin{array}{l} \text{AMPK}\left(\text{ulk1!1,T172\textasciitilde{}P,ST\textasciitilde{}0}\right)\text{.ULK1}\left(\text{stampk!1,S778\textasciitilde{}0}\right)\text{-> \textbackslash{}}\\ \text{AMPK}\left(\text{ulk1!1,T172\textasciitilde{}P,ST\textasciitilde{}0}\right)\text{.ULK1}\left(\text{stampk!1,S778\textasciitilde{}P}\right)\;\text{p8} \end{array}$$ (11B)

where p7 and p8 are (pseudo first-order) rate constants. Each rule indicates that AMPK kinase activity requires 1) phosphorylation at T172 in the kinase domain of the PRKAA2 isoform of the α subunit (or equivalently, phosphorylation at T183 in the kinase domain of the PRKAA1 isoform of the α subunit), 2) the absence of phosphorylation at S/T residues in the α subunit outside the kinase domain, and 3) association of AMPK with ULK1, which is mediated by the ulk1 and stampk sites in AMPK and ULK1, respectively. Furthermore, AMPK-mediated phosphorylation of S317 in ULK1, but not S778, requires that ULK1 be free of RPTOR (i.e., the straptor site in ULK1 must be free). The reason for this constraint is that RPTOR appears to mask S317 when bound to ULK1, which would prevent phosphorylation of S317 by AMPK. RPTOR interacts with the PS domain in ULK1, which contains S317 [10,55].

#### AMPK phosphorylates RPTOR at S792

We represent AMPK-mediated inhibitory phosphorylation of RPTOR at S792 using a unidirectional rule, as follows:

AMPK(T172~P)+RPTOR(S792~0)->AMPK(T172~P)+RPTOR(S792~P)p9 (12)

where p9 is a (pseudo first-order) rate constant. Equation (12) indicates that AMPK*, regardless of the phosphorylation status of S/T residues in the α subunit outside the kinase domain (i.e., regardless of the internal state of the ST component of AMPK), is able to phosphorylate RPTOR at S792. Note that AMPK and RPTOR are not required to form a complex for AMPK-mediated phosphorylation of RPTOR to occur. Thus, we are assuming that the enzymatic reactions defined by the rule of Equation (12) are occurring in the regime of substrate limitation, far from enzyme saturation.

#### Dephosphorylation of S758 in ULK1

We represent dephosphorylation of S758 in ULK1 as follows:

ULK1(S758~P)->ULK1(S758~0)u0 (13)

where u0 is a (pseudo first-order) rate constant. In most cases, for simplicity, rules for dephosphorylation reactions are assigned the dephosphorylation rate constant u0. We assume that dephosphorylation reactions are mediated by constitutively active (i.e., unregulated) phosphatases, which are taken to be present in excess. This rule exemplifies our treatment of phosphatases, which generally are not as well characterized as kinases.

#### Dephosphorylation of S65 and T70 in EIF4EBP1

We represent dephosphorylation of S65 and T70 in EIF4EBP1 as follows:

EIF4EBP1(S65_T70~P)->EIF4EBP1(S65_T70~0)u0 (14)

where u0 is a (pseudo first-order) rate constant. This rule is similar to that of Equation (12).

#### Dephosphorylation of S792, S855 and S859 in RPTOR

We represent dephosphorylation of S792, S855 and S859 in RPTOR as follows:

RPTOR(S792~P)->RPTOR(S792~0)u1 (15A)

RPTOR(S855_S859~P)->RPTOR(S855_S859~0)u0 (15B)

RPTOR(S855_S859~PP)->RPTOR(S855_S859~P)2*u0 (15C)

where u0 and u1 are (pseudo first-order) rate constants. We assume that S792 in RPTOR is dephosphorylated more slowly than typical sites considered in the model. Consequently, the rule of Equation (15A) is assigned the rate constant u1 (instead of u0). The rate constant in the rule of Equation (15C) is specified as twice that for the rule of Equation (15B) because the PP to P state transition for site S855_S859 is expected to occur twice as fast as the P to 0 transition.

#### Dephosphorylation of S/T residues in AMBRA1

We represent dephosphorylation of S/T residues in AMBRA1 as follows:

AMBRA1(ST~P)->AMBRA1(ST~0)u0 (16)

This rule is similar to that of Equation (13).

#### Dephosphorylation of ST residues in AMPK

We represent dephosphorylation of S/T residues in AMPK as follows:

AMPK(ST~P)->AMPK(ST~0)u2 (17)

where u2 is a (pseudo first-order) rate constant. We assume that S/T residues in AMPK are dephosphorylated more slowly than typical sites considered in the model, so the rule of Equation (17) is assigned the rate constant u2 (instead of u0). This rule is otherwise similar to that of Equation (13).

#### Dephosphorylation of S317 and S778 in ULK1

We represent dephosphorylation of S317 and S778 in ULK1 as follows:

$$\begin{array}{l} \text{ULK1}\left(\text{straptor,S317\textasciitilde{}P}\right)\text{->ULK1}\left(\text{straptor,S317\textasciitilde{}0}\right)\;\text{u0} \end{array}$$ (18A)

ULK1(S778~P)->ULK1(S778~0)u0 (18B)

These rules are similar to that of Equation (13) except that the rule of Equation (18A) imposes a constraint on dephosphorylation of S317 in ULK1. Namely, dephosphorylation of this site is only allowed to occur when ULK1 is free of RPTOR (or equivalently, the straptor site in ULK1 is free). This requirement is introduced because S317 overlaps with the straptor site. We assume that S317 cannot be phosphorylated or dephosphorylated when RPTOR is in contact with the straptor site.

#### Dephosphorylation of S792 in RPTOR

We represent dephosphorylation of S792 in RPTOR as follows:

RPTOR(S792~P)->RPTOR(S792~0)u0 (19)

This rule is similar to that of Equation (13).

#### Parameters

Even though the interactions considered in our model are fairly well established, information about the quantitative factors that influence these interactions (e.g., rate constants and concentrations) is scarce. To cope with this knowledge gap, we assigned values to the parameters of the model, which are summarized in Table 1, somewhat arbitrarily within ranges deemed to be reasonable, and we eschewed fine-tuning of parameter values. Thus, for each rate constant, we only specified an order of magnitude (i.e., we only used a single significant digit, 1). To offset uncertainty about parameter values, we focused on qualitative system behavior through bifurcation analyses and we also performed a systematic parameter sensitivity analysis. Our rationale for setting parameter values is further explained below.

#### Rate constants for dephosphorylation reactions

The model includes 10 rules for dephosphorylation reactions, which are given by Equations (13)–(19). Each of these rules is associated with a rate constant and, in accordance with the conventions of BNGL, a mass-action rate law that has this rate constant as its only parameter.

We associated 8 of the 10 rules for dephosphorylation with a common rate constant, *u*_0_. We set the nominal value of *u*_0_ at 10^−2^ s^−1^ (Table 1). This setting essentially establishes the characteristic response time of the system, which is on the order of minutes [14]. Shang et al. [14] observed that inhibitory phosphorylation of ULK1 decreased over a period of minutes after activation of autophagy through starvation or rapamycin treatment.

We associated the rule of Equation (15A), which characterizes dephosphorylation of S792 in RPTOR, with the rate constant *u*_1_. We set the nominal value of *u*_1_ at 10 times less than the value of *u*_0_ (Table 1). This setting is motivated by interactions of phosphorylated S792 (pS792) with 14–3–3 proteins, which are known to protect pS792 from dephosphorylation [11]. Thus, by setting *u*_1_ = 0.1 *u*_0_, we are implicitly considering the 14–3–3 binding partners of pS792 and accounting for shielding of pS792 from phosphatases by these binding partners.

We associated the rule of Equation (15A), which characterizes dephosphorylation of the serine/threonine residues in AMPK that are substrates of ULK1, with the rate constant *u*_2_. We set the nominal value of at *u*_2_ at 100 times less than the value of *u*_0_ (Table 1). By setting *u*_2_ = 0.01 *u*_0_, we are assuming that the time scale of negative feedback from ULK1 to AMPK is much slower than the time required to respond to a stress stimulus. A slower time scale is necessary to allow for a period of autophagy, i.e., a period during which ULK1 kinase activity is sustained, which is presumably required for autophagosome production. Fast negative feedback would quickly shut off ULK1 activity after a stress stimulus, potentially severely limiting autophagy.

#### Rate constants for phosphorylation reactions

The model includes 10 rules for phosphorylation reactions, which are given by Equations (6)–(12). Each rule is associated with a rate constant.

We assigned the phosphorylation rate constants *p*_1_, *p*_2_, *p*_3_, *p*_4_, *p*_7_, and *p*_8_ a common nominal value, 10 s^−1^ (Table 1). The value assigned to these rate constants is high (cf. the value assigned to *u*_0_, 0.01 s^−1^) because each of these rate constants characterizes a pseudo first-order reaction in which an enzyme and one of its substrates are colocalized within a protein complex, i.e., confined together within a small reaction volume.

The remaining phosphorylation rate constants, *p*_5_, *p*_6_, and *p*_9_, characterize pseudo second-order reactions. We assume that each of these (overall) reactions corresponds to a Michaelis-Menten reaction scheme operating in the regime of substrate limitation, far from enzyme saturation. Thus, each second-order rate constant corresponds to a ratio of the form *k*_cat_/*K*_M_.

We set the nominal value of *p*_5_ at 10^−4^ (molecule/cell)^−1^s^−1^ (Table 1). This setting is arbitrary and has little influence on predicted system behavior (Fig. 8) because *p*_5_ characterizes ULK1-mediated phosphorylation of AMBRA1. In the model, AMBRA1 phosphorylation serves as a reporter of ULK1 kinase activity.

We set the nominal value of *p*_6_, which characterizes ULK1-mediated inhibitory phosphorylation of AMPK (i.e., the strength of negative feedback), at 10^−6^ (molecule/cell) ^−1^s^−1^ (Table 1). In initial exploratory simulations, this parameter setting was found to allow for both robust activation of autophagy and nearly complete inhibition of autophagy through negative feedback from ULK1 to AMPK. It should be noted that ULK1 is responsible for phosphorylating multiple serine/threonine residues in AMPK, which in the model are lumped together. Thus, *p*_6_ is an effective rate constant.

We set the nominal value of *p*_9_, which characterizes AMPK-mediated inhibitory phosphorylation of RPTOR, at 0 (Table 1). In initial exploratory simulations, we found that phosphorylation of S792 in RPTOR by AMPK limits the region of bistability (in the case without negative feedback from ULK1 to AMPK) and the region of oscillatory behavior (in the case with negative feedback from ULK1 to AMPK) in the parameter space of inputs. Thus, we set the nominal value of *p*_9_ to 0, and we gave non-zero values of *p*_9_ special attention in our evaluation of the model. In other words, we performed bifurcation analyses in which *p*_9_ was a bifurcation parameter (Fig. 7).

#### Rate constants for association and dissociation reactions

The model includes two reversible rules and seven unidirectional rules for association/dissociation reactions, which are given by Equations (1)–(5). The rules are associated with a total of 11 rate constants. Nominal values for the rate constants of association and dissociation reactions (Table 1) were set with the considerations noted below in mind.

We selected values for *a*_1_, *d*_1_, *a*_2_, and *d*_2_ such that the affinity of rapamycin* for MTOR is 10-fold greater than the affinity of RPTOR for MTOR. In other words, we selected values for these rate constants such that *a*_1_/ *d*_1_ = 10 *a*_2_/*d*_2_. This approach is motivated by the ability of rapamycin to disrupt binding of RPTOR to MTOR [61].

In initial exploratory simulations, we found that the rate constants governing ULK1 interactions with AMPK and RPTOR affect the bias of a cell toward translation (or autophagy). An increase in the affinity of ULK1 for AMPK makes a cell more prone for autophagy. Conversely, an increase in the affinity of ULK1 for RPTOR makes a cell more prone for translation. Thus, we selected values for *a*_3_, *d*_3_, *a*_5_, and *d*_5_ that yield roughly balanced propensities for translation and autophagy.

The value of *d*_3,max_ was set such that *d*_3,max_ is much greater than *d*_3_: we set *d*_3,max_ = 100 *d*_3_. Recall that RPTOR, when phosphorylated at particular residues, dissociates from ULK1 faster than it otherwise would (cf. Equations (3B) and (3C)). The rate constant *d*_3_ characterizes slow dissociation of RPTOR from ULK1 and the rate constant *d*_3,max_ characterizes fast dissociation of RPTOR from ULK1.

Finally, we selected values for *a*_4_ and *d*_4_, which govern interaction of EIF4EBP1 with RPTOR, so as to avoid sequestration of MTORC1 by EIF4EBP1. Recall that EIF4EBP1 is included in the model simply to serve as a reporter of MTORC1 kinase activity.

#### Concentrations

In U2OS cells, for example, the total cellular abundances of ULK1 and AMBRA1 are on the order of 10^4^ copies per cell, whereas the total cellular abundances of AMPK, MTOR, RPTOR, and EIF4EBP1 are far greater, roughly on the order of 10^6^ copies per cell [62]. We assume that all proteins are present at effective concentrations of around 10^4^ copies per cell. This assumption is introduced to account for sequestration of AMPK and MTORC1 away from ULK1 by their binding partners not considered in the model, as well as the different spatial distributions of ULK1 and MTORC1 (punctate and localized to membrane sites of autophagosome formation in the case of ULK1 vs. cytosolic and lysosomally localized in the case of MTORC1) [40,63]. Comparable effective levels of ULK1 and MTORC1 is consistent with the observation that ULK1, through phosphorylation of RPTOR, is able to relieve inhibition of autophagy by MTORC1, which seems unlikely if the entire pool of MTORC1 in a cell is available to interact with ULK1.

We set concentrations or copy numbers as follows. We set the abundances of MTOR and RPTOR to be equal, at 2×10^4^ copies per cell. This setting was guided by the 1:1 stoichiometry of MTOR and RPTOR in MTORC1. We set the abundance of ULK1 at half the abundance of MTOR, 10^4^ copies per cell. It is important for ULK1 to be less abundant than MTORC1 so that MTORC1 is able to fully repress ULK1 while some fraction of MTORC1 remains available to interact with EIF4EBP1, which is included in the model as a reporter for translation activity. Full repression of ULK1 seems reasonable because autophagy can be repressed to undetectable levels when cells are not stressed. The levels of EIF4EBP1 and AMBRA1 were set at 10^4^ copies per cell. These settings are largely inconsequential for predicted system behavior, because there is no feedback from these proteins to other molecules that are considered in the model. Recall that EIF4EBP1 and AMBRA1 are included in the model only for the purpose of providing readouts of MTORC1 and ULK1 activities, which presumably correlate with translation and autophagy levels. We treat the levels of AMPK* and rapamycin* as controllable inputs. Recall that AMPK* is distinct from total AMPK and active AMPK.

### Simplifications

Our model omits several known interactions among the proteins of interest. Furthermore, AMPK, RPTOR, and ULK1 each contain more sites of phosphorylation than are included in the model. These interactions and sites, which are discussed below, were omitted to keep the model as simple as possible.

Some of the omitted sites have roles similar to sites included in the model. In RPTOR, S863 and S877 are additional substrates of ULK1 that are involved in ULK1-mediated inhibition of MTORC1 kinase activity [15]. In ULK1, S479 and S556 appear to play a role analogous to that of S758; in other words, they appear to be additional substrates of MTORC1 involved in MTORC1-mediated inhibition of ULK1 kinase activity [14]. Besides S317 and S778, ULK1 contains additional sites phosphorylated by AMPK, including S467, S555, S637, and T659, which are involved in activation of ULK1 [64,65,66]. The model does not individually consider the multiple serine/threonine substrates of ULK1 that are found in the catalytic α subunit of AMPK, which are involved in negative feedback regulation of AMPK by ULK1 [24]. Instead, these sites are lumped together, i.e., they are treated as a single site.

One of the omitted sites has an unclear functional role. Shang et al. [14] reported that ULK1 is phosphorylated at S638 by both AMPK and MTORC1. This finding is enigmatic because phosphorylation of other sites in ULK1 by MTORC1 has an inhibitory effect on ULK1 kinase activity, whereas phosphorylation of other sites in ULK1 by AMPK has an activating effect. Thus, the role of S638 may be distinct from that of other AMPK and MTORC1 substrates. Because the role of this site is unclear, we did not include S638 in the model.

In formulating the model, we focused on interactions among AMPK, MTORC1, and ULK1, because this triad of kinases is recognized as playing a critical role in regulation of autophagy and translation [50]. However, the triad network is embedded within a larger regulatory network. Below, we call attention to complicating features of this larger network, which are beyond the scope of this study but that are, under certain conditions, likely to affect the behavior of the triad network. It is important to call attention to these complicating features to recognize the limitations of the present study.

Recently, Nazio et al. [38] reported positive feedback from AMBRA1 to ULK1. In this feedback loop, ULK1-activated AMBRA1 interacts with the E3 ubiquitin ligase TRAF6, which enables TRAF6 to mediate the attachment of K63-linked chains of ubiquitin to ULK1, which stabilizes ULK1 kinase activity. Interestingly, AMBRA1-mediated ubiquitylation of ULK1 is antagonized by MTORC1-mediated phosphorylation of S52 in AMBRA1, which provides an additional (indirect) avenue for MTORC1 to inhibit ULK1 [38], which could strengthen the robustness of oscillatory behavior. In the model, we included AMBRA1 only as a reporter of ULK1 kinase activity, essentially considering a situation where, for simplicity, positive feedback from AMBRA1 to ULK1 is abrogated. Likewise, we did not consider negative feedback regulation of MTOR by amino acids generated through autophagy [28], which is similar to but distinct from negative feedback regulation of AMPK by ULK1.

In addition to being a component of MTORC1, MTOR is a component of a second complex (MTORC2), which is distinguished by the subunit RICTOR. Following growth factor simulation, MTORC2 promotes full activation of AKT, which subsequently signals downstream to MTORC1 [67]. Although rapamycin is largely selective for MTORC1, on long time scales (e.g., 24 hours), rapamycin causes dissociation of MTORC2 [68]. Thus, it should be understood that in our analyses we are considering responses to rapamycin on relatively short time scales.

As mentioned earlier, our model simplifies the regulation of MTORC1 by AMPK. It is well established that AMPK positively regulates TSC2 (within the TSC complex, which also consists of TSC1 and TBC1D7) via phosphorylation, which in turn suppresses the activity of the small GTPase RHEB, a direct activator of MTORC1 [45]. For simplicity, our model considers only the more direct route by which AMPK regulates MTORC1, which is achieved through phosphorylation of RPTOR.

### Simulations, bifurcation analysis, and sensitivity analysis

We performed simulations using a deterministic method available within BioNetGen [53]. This method, which is an indirect method, involves first processing the rules of the model specification (S1 File) to obtain the reaction network implied by the rules, as well as the corresponding ordinary differential equations (ODEs) for mass-action kinetics. The reaction network consists of 173 chemical species and 6,581 unidirectional reactions. (The size of the reaction network reflects the number of protein phosphoforms and protein complexes that can arise from the interactions represented by the rules of the model.) BioNetGen’s built-in ODE solver, CVODE from the SUNDIALS package [69], was then used to numerically integrate the ODEs, using default settings. The steps described above were performed automatically by BioNetGen and invoked using point-and-click commands available within RuleBender [70], which provides a graphical user interface for accessing BioNetGen’s capabilities.

Each one-dimensional bifurcation analysis was performed numerically as follows. We added a rule to the model specification (S1 File) that has the form 0->p k_epsilon or p->0 k_epsilon, where 0 is a source or sink, p is the bifurcation parameter, and k_epsilon is sufficiently small. The added rule has the effect of either very slowly increasing or very slowly decreasing the value of the bifurcation parameter (e.g., the level of AMPK* or rapamycin*), such that other processes are in a pseudo steady state, which slowly changes, as the bifurcation parameter is varied. Simulations were performed with the bifurcation parameter gradually increasing (from a sufficiently small value) and also gradually decreasing (from a sufficiently large value) to find stable steady states and the lower and upper bounds of stable limit cycles for values of the bifurcation parameter of interest. Two-dimensional bifurcation analyses were performed similarly. We allowed one parameter to vary while holding the other at a fixed value. We then repeated this procedure for different values of the second parameter. The numerical methods described above, in contrast to continuation methods implemented in software tools such as AUTO and MatCont, do not allow for characterization of unstable steady states or unstable limit cycles, but with these methods, it is possible to analyze the (large) system of ODEs of interest, for which continuation methods are prohibitively inefficient.

In the sensitivity analysis of Fig. 8, we performed two series of one-dimensional bifurcation analyses for each of the 22 parameters considered. In the first of the two series, the bifurcation parameter was the level of AMPK*. In all cases, we considered levels of AMPK* from 0 to 10^6^ copies per cell. In the second of the two series, the bifurcation parameter was the level of rapamycin*. In all cases, we considered levels of rapamycin* from 0 to 10^5^ copies per cell. In each series, we considered 17 different values for one of the 22 parameters: we set *P*_m_ = *P*_m,0_ ×10^n/4^ for *n* = −8,…,+8, where *m* is an index, in the range [1,…, 22], that identifies the parameter and *P*_m,0_ is the nominal value of the parameter given in Table 1. The results from each series of bifurcation analyses were used to find the range of values for parameter *P*_m_ for which the following pattern, illustrated in Fig. C (S2 File), holds true: 1) low AMBRA1 phosphorylation and high EIF4EBP1 phosphorylation at low values of the bifurcation parameter, 2) oscillations in AMBRA1 and EIF4EBP1 phosphorylation at intermediate values of the bifurcation parameter, and 3) high AMBRA1 phosphorylation and low EIF4EBP1 phosphorylation at high values of the bifurcation parameter. The ranges so found are reported in Fig. 8.

## Supporting Information

S1 File Executable model specification. This plain-text file provides an executable model specification, which can be processed by BioNetGen. The filename extension should be changed to “.bngl” for processing by BioNetGen.
(TXT)

S2 File Figs. A, B and C. This PDF file combines Figs. A, B and C. **Fig. A. Phosphorylation of AMBRA1, EIF4EBP1, and sites in RPTOR and ULK1 in translation and autophagy states of the system**. When AMBRA1 phosphorylation is high, EIF4EBP1 phosphorylation is low. This pattern of phosphorylation defines an autophagy state of the system. Conversely, when AMBRA1 phosphorylation is low, EIF4EBP1 phosphorylation is high. This pattern of phosphorylation defines a translation state of the system. The bars shown here indicate the fractional phosphorylation levels of various protein sites considered in the model for a condition where bistability exists. Blue bars correspond to a translation state, which is one of two stable steady states that can be realized for an AMPK* level of 30,000 copies per cell, no rapamycin*, and other parameters set at their nominal values (Table 1). Red bars correspond to an autophagy state, which is the other realizable stable steady state for the inputs and parameter values indicated above. Fig. B. Durations of the autophagy and translation phases in the oscillatory regime depend on AMPK* and rapamycin* levels. We define the autophagy (translation) phase of an oscillation as the period during which more (less) than half of all AMBRA1 is phosphorylated. (A) The red (blue) curve reports the duration of the autophagy (translation) phase as a function of AMPK* level. The level of rapamycin* is zero. (B) The red (blue) curve reports the duration of the autophagy (translation) phase as a function of rapamycin* level. The level of AMPK* is 30,000 copies per cell. In both panels, the parameters considered in Table 1 are set at their nominal values. **Fig. C. Examples of response patterns to varying levels of a stress input**. Each panel shows the response of the system to a slowly increasing level of AMPK*. The vertical axis reports the fraction of AMBRA1 that is phosphorylated. The horizontal axis reports AMPK* level. In all cases, rapamycin* is zero and parameters are set at their nominal values, except as indicated. (A) The blue curve corresponds to the case where the value of *p*_4_ is 100-fold above its nominal value, and the red curve corresponds to the case where the value of *p*_4_ is 100-fold below its nominal value. In both cases, the qualitative pattern of response is the same: low AMBRA1 phosphorylation at low AMPK* levels, oscillations at intermediate AMPK* levels, and high AMBRA1 phosphorylation at high AMPK* levels. Thus, system behavior is robust to changes in the value of *p*_4_, the rate constant for phosphorylation of S855 and S859 in RPTOR. (B) As illustrated here, system behavior is less robust to changes in the value of *p*_6_, the rate constant for inhibitory phosphorylation of AMPK. When *p*_6_ is 10^3/4^ times its nominal value, the characteristic pattern of response is conserved (black curve). However, when *p*_6_ is 10^4/4^ times its nominal value, the system responds monotonically to increasing AMPK* level over the range of AMPK* levels considered (green curve). (C) Similarly, when *p*_6_ is 10^−2/4^ times its nominal value, the characteristic pattern of response is conserved (black curve). However, when *p*_6_ is 10^−3/4^ times its nominal value, the system switches abruptly from low AMBRA1 phosphorylation to high AMBRA1 phosphorylation without exhibiting oscillations (green curve). The results of panels B and C indicate that system responses to changes in the level of AMPK* are robust to variations of *p*_6_ that range from 10^−2/4^ to 10^4/4^ times the nominal value of *p*_6_ (cf. red bar corresponding to *p*_6_ in Fig. 8).
(PDF)
