# Supplementary material for: Computational Analysis of an Autophagy/Translation Switch Based on Mutual Inhibition of MTORC1 and ULK1
Source: PLoS One. 2015 Mar 11;10(3):e0116550. doi: 10.1371/journal.pone.0116550 (PMC4356596; doi:10.1371/journal.pone.0116550)
Supplement: S2 File — This PDF file combines Figs. A, B and C. Fig. A. Phosphorylation of AMBRA1, EIF4EBP1, and sites in RPTOR and ULK1 in translation and autophagy states of the system. When AMBRA1 phosphorylation is high, EIF4EBP1 phosphorylation is low. This pattern of phosphorylation defines an autophagy state of the system. Conversely, when AMBRA1 phosphorylation is low, EIF4EBP1 phosphorylation is high. This pattern of phosphorylation defines a translation state of the system. The bars shown here indicate the fractional phosphorylation levels of various protein sites considered in the model for a condition where bistability exists. Blue bars correspond to a translation state, which is one of two stable steady states that can be realized for an AMPK* level of 30,000 copies per cell, no rapamycin*, and other parameters set at their nominal values (Table 1). Red bars correspond to an autophagy state, which is the other realizable stable steady state for the inputs and parameter values indicated above. Fig. B. Durations of the autophagy and translation phases in the oscillatory regime depend on AMPK* and rapamycin* levels. We define the autophagy (translation) phase of an oscillation as the period during which more (less) than half of all AMBRA1 is phosphorylated. (A) The red (blue) curve reports the duration of the autophagy (translation) phase as a function of AMPK* level. The level of rapamycin* is zero. (B) The red (blue) curve reports the duration of the autophagy (translation) phase as a function of rapamycin* level. The level of AMPK* is 30,000 copies per cell. In both panels, the parameters considered in Table 1 are set at their nominal values. Fig. C. Examples of response patterns to varying levels of a stress input. Each panel shows the response of the system to a slowly increasing level of AMPK*. The vertical axis reports the fraction of AMBRA1 that is phosphorylated. The horizontal axis reports AMPK* level. In all cases, rapamycin* is zero and parameters are set at their no [file pone.0116550.s002.pdf]

Supplementary File S2: Figures A–C

Computational Analysis of an Autophagy/Translation Switch Based on Mutual Inhibition of mTORC1 and ULK1

Paulina Szymańska, Katie R. Martin, Jeffrey P. MacKeigan, William S. Hlavacek, and Tomasz Lipniacki

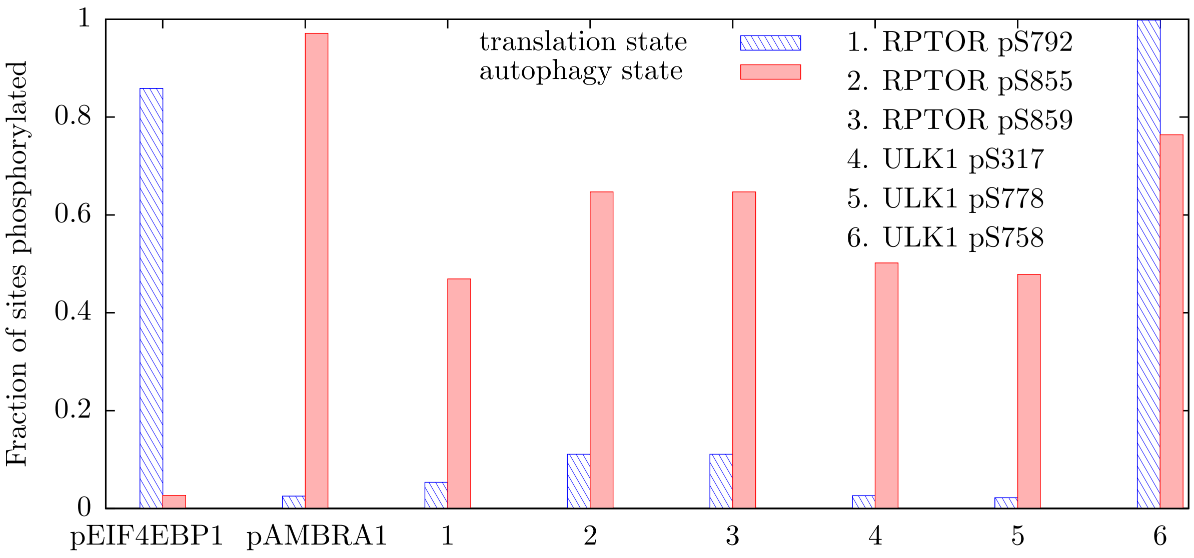

Figure A. Phosphorylation of AMBRA1, EIF4EBP1, and sites in RPTOR and ULK1 in translation and autophagy states of the system. When AMBRA1 phosphorylation is high, EIF4EBP1 phosphorylation is low. This pattern of phosphorylation defines an autophagy state of the system. Conversely, when AMBRA1 phosphorylation is low, EIF4EBP1 phosphorylation is high. This pattern of phosphorylation defines a translation state of the system. The bars shown here indicate the fractional phosphorylation levels of various protein sites considered in the model for a condition where bistability exists. Blue bars correspond to a translation state, which is one of two stable steady states that can be realized for an AMPK* level of 30,000 copies per cell, no rapamycin*, and other parameters set at their nominal values (Table 1). Red bars correspond to an autophagy state, which is the other realizable stable steady state for the inputs and parameter values indicated above.

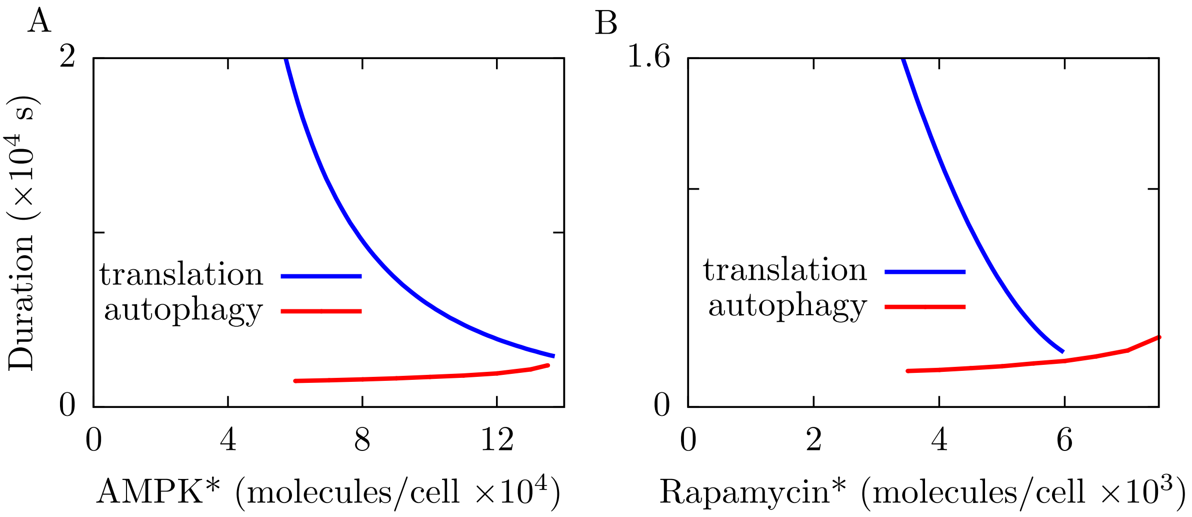

Figure B. Durations of the autophagy and translation phases in the oscillatory regime depend on AMPK* and rapamycin* levels. We define the autophagy (translation) phase of an oscillation as the period during which more (less) than half of all AMBRA1 is phosphorylated. (A) The red (blue) curve reports the duration of the autophagy (translation) phase as a function of AMPK* level. The level of rapamycin* is zero. (B) The red (blue) curve reports the duration of the autophagy (translation) phase as a function of rapamycin* level. The level of AMPK* is 30,000 copies per cell. In both panels, the parameters considered in Table 1 are set at their nominal values.

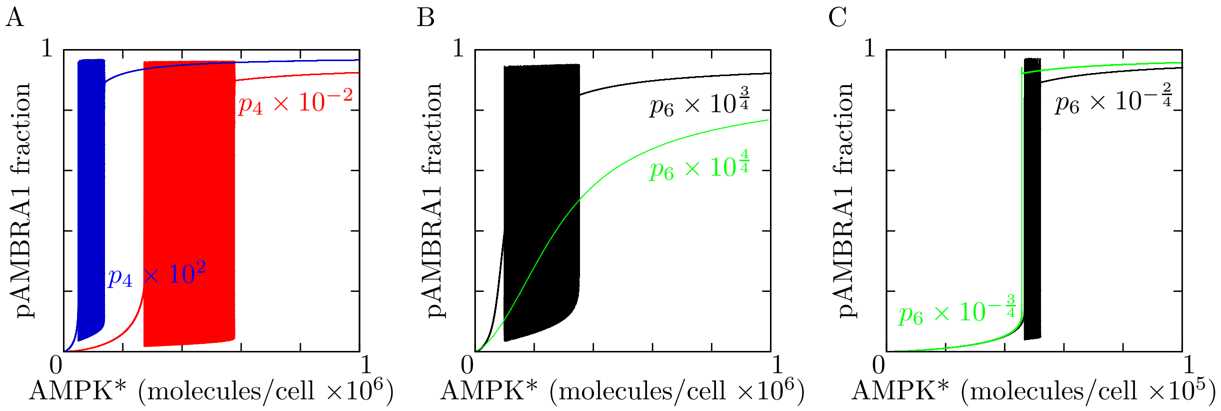

Figure C. Examples of response patterns to varying levels of a stress input. Each panel shows the response of the system to a slowly increasing level of AMPK*. The vertical axis reports the fraction of AMBRA1 that is phosphorylated. The horizontal axis reports AMPK* level. In all cases, rapamycin* is zero and parameters are set at their nominal values, except as indicated. (**A**) The blue curve corresponds to the case where the value of p_4 is 100-fold above its nominal value, and the red curve corresponds to the case where the value of p_4 is 100-fold below its nominal value. In both cases, the qualitative pattern of response is the same: low AMBRA1 phosphorylation at low AMPK* levels, oscillations at intermediate AMPK* levels, and high AMBRA1 phosphorylation at high AMPK* levels. Thus, system behavior is robust to changes in the value of p_4, the rate constant for phosphorylation of S855 and S859 in RPTOR. (**B**) As illustrated here, system behavior is less robust to changes in the value of p_6, the rate constant for inhibitory phosphorylation of AMPK. When p_6 is $10^{3/4}$ times its nominal value, the characteristic pattern of response is conserved (black curve). However, when p_6 is $10^{4/4}$ times its nominal value, the system responds monotonically to increasing AMPK* level over the range of AMPK* levels considered (green curve). (**C**) Similarly, when p_6 is $10^{-2/4}$ times its nominal value, the characteristic pattern of response is conserved (black curve). However, when p_6 is $10^{-3/4}$ times its nominal value, the system switches abruptly from low AMBRA1 phosphorylation to high AMBRA1 phosphorylation without exhibiting oscillations (green curve). The results of panels B and C indicate that system responses to changes in the level of AMPK* are robust to variations of p_6 that range from $10^{-2/4}$ to $10^{4/4}$ times the nominal value of p_6 (cf. red bar corresponding to p_6 in Figure 8).
